# ‘Blue-lighting’ seizure-related needs in care homes: a retrospective analysis of ambulance call-outs for seizures in North West England (2014–2021), their management and costs, with community comparisons

**DOI:** 10.1136/bmjopen-2024-089126

**Published:** 2024-11-13

**Authors:** Adam J Noble, Steven Lane, Peter Lloyd, Beth Morris, Steve Bell, Tom Shillito, Pete Dixon, Anthony Guy Marson

**Affiliations:** 1 Department of Public Health, Policy and Systems, University of Liverpool, Liverpool, UK; 2 Health Data Science, University of Liverpool, Liverpool, UK; 3 Public Contributor, Liverpool, UK; 4 Medical Directorate, North West Ambulance Service NHS Trust, Bolton, UK; 5 Epilepsy Action, Leeds, UK; 6 Department of Primary Care and Mental Health, University of Liverpool, Liverpool, UK; 7 Molecular and Clinical Pharmacology, University of Liverpool, Liverpool, UK

**Keywords:** Epilepsy, Nursing Homes, Dementia, Health Services for the Aged, Emergency Departments

## Abstract

**Abstract:**

**Objectives:**

With a projected rise in care home residency and the disproportionate impact of epilepsy and seizures on older adults, understanding seizure-related needs in this population is crucial. Data silos and inconsistent recording of residence status make this challenging. We thus leveraged ambulance data to investigate seizure call-out incidence, characteristics, management and costs in care homes compared with the wider community.

**Design:**

Retrospective analysis of dispatch data from a regional English ambulance service over four 9-month periods between 2014/2015 and 2021/2022. Suspected seizures in adults (≥16 years) were identified, with data on location, patient age, severity and management extracted. Incidence rates, trends over time and case characteristics were compared. Costs of ambulance response were estimated, and factors influencing emergency department (ED) conveyance were analysed using logistic regression.

**Setting:**

North West Ambulance Service National Health Service Trust, serving an adult population of ~5.5 million.

**Participants:**

Dispatch data for 98 752 suspected seizure cases.

**Results:**

Care homes, accommodating ~0.8% of the regional population, accounted for 7.2% of seizure call-outs. Incidence was higher in care homes than the wider community (55.71 vs 5.97 per 1000 person/year in 2021/2022) and increased over time. Care home cases peaked around 8:00–9:00. Despite similar or lower severity, they had a higher ED conveyance rate (78.3% vs 70.6%). Conveyance likelihood was influenced by factors beyond severity: reduced in homes specialising in learning disabilities (adjusted OR=0.649) and increased in homes with nursing provision (adjusted OR=1.226). Care homes accounted for 7.26% of the £24 million cost.

**Conclusions:**

This study highlights the growing seizure-related needs in care homes. Despite similar severity, most cases result in ED conveyance. Future research should examine the appropriateness and implications of these transfers, ensuring specialist services support the care home population effectively.

**STRENGTHS AND LIMITATIONS OF THIS STUDY**

- Examination of data from four different time periods permitted an examination of temporal changes in the incidence of call-outs for suspected seizures, both inside and outside care homes.
- Narrow CIs surrounding the estimates enhance the reliability and precision of the findings.
- Findings are derived from a single regional service, potentially limiting generalisability.
- The use of routine data, which was pseudo-anonymised and sometimes missing, prevented certain analyses and introduced potential data quality issues.

## Background

 Epilepsy is a common neurological disorder (UK prevalence ~1%).^1^ Who it affects is changing.^2^ An ageing population, combined with epilepsy’s links to dementia, stroke and brain degeneration, means incidence in Europe is now highest in those aged ≥75.^3^ Acute symptomatic seizures also increase with age.^4^ It is important to identify environments where needs might be increasing and explore the adequacy of patient support. This information is fundamental to service planning, not least to allow specialist adult epilepsy services—who tend to be responsible for initial diagnosis and treatment planning of people aged ≥16^5^—to plan and understand the adequacy of their provision.

One environment where needs might be changing is care homes. Residents are theoretically at heightened risk—most (82%) are aged ≥65^6^ and ~70% have dementia.^7^ Currently, ~370 000 (0.6%) of England’s population reside in a care home.^8^ As in other countries, this number is set to rapidly rise (by 150% by 2031 according to some projections).^9^

England’s care regulator defines a care home as a facility providing personal care and accommodation,^10^ excluding establishments like assisted living or specialist educational facilities.^10^ Homes vary in size, ownership, specialism, residents’ needs and staffing. One-third have registered nursing provision,^11^ with their residents generally being in poorer health.^11^ In the remainder, support is provided by care assistants, with clinical input provided by visiting health professionals. Other differences in staffing likely exist between homes but are hard to capture, as care homes are not required to publicise staffing profiles, nor adhere to specific staff numbers, skill mix ratios^12^ or regulations regarding engagement with visiting health professionals.^13 14^

Currently, there is minimal information on epilepsy and seizures in the care home population, likely due to data silos and inconsistent resident status recording.^15^ One information source less affected by these issues is ambulance data. Their dispatch records capture both the nature of cases and where they occur.

England’s ambulance service is comprised of 10 regional organisations. Examining dispatch data from one of them over various time periods could be enlightening. While it cannot determine the exact number of seizures occurring in care homes, it can indicate how many result in ambulance calls. This allows for an assessment of demand, change and the proportion of care homes seeking assistance.

Another reason for analysing such data is differences exist in the presentation and aetiology of epilepsy and seizures in older persons compared with younger persons.^16 17^ Ambulance records capture key information about cases. They might thus provide insights into how seizures manifest in the care home population and implications for practice.

Finally, dispatch data captures how cases are managed by the ambulance service. This is relevant because suspected seizure cases in the wider community (outside of care homes) are often ‘over conveyed’ to emergency departments (EDs). These frequently involve individuals with established diagnoses (typically epilepsy or psychogenic conditions) experiencing uncomplicated seizures.^18^,^20^ In 90% of cases, the seizure has self-terminated before the paramedic arrives, and breathing is normal.^18^ Factors, such as the person being in public, alone and without medical information to contextualise their presentation, appears to create momentum for conveyance.^21 22^ Visiting ED after an uncomplicated seizure is not recommended,^23^ not risk-free,^24 25^ and is costly to the National Health Service (NHS).^18 26^ Evidence from a range of sources, including discrete choice experiments, indicates such visits are also not typically preferred by with adults living with epilepsy or their significant others.^27 28^ While dispatch data will not allow for assessing the appropriateness of care home case management, it can permit insights into their management, explore influencing factors and estimate the direct NHS costs.

We systematically searched the literature and found only one study that has considered the role of care homes in seizure-related ambulance use (online supplemental material 1). Magnusson and Zelano^29^ reported 11% of seizure-related ambulance cases in Gothenburg arose from ‘residential homes’. While important, this study was small and did not examine changes over time, provide incidence rates, estimate cost or compare care home and wider community cases.

In this present study, we, therefore, analysed dispatch data from England’s North-West Regional Ambulance Service (NWAS), which serves a population of around ~5.5 million. We sought to answer the following:

1. What is the incidence of suspected seizure cases presenting to the ambulance service from care homes, is it changing, and how does this compare to those in the wider community?
2. What proportion of all ambulance seizure cases do care homes account for?
3. What are the characteristics of care home cases, and how do they compare to those in the wider community?
4. How does the ambulance service manage cases within care homes, and how does this compare to those in the wider community?
5. What are the typical and aggregate direct costs to the NHS associated with ambulance response to cases within care homes, and how do these compare to those for wider community cases?
6. What case characteristics are associated with whether an attending ambulance conveys a care home seizure to ED, and how does this compare to those in the wider community?

To provide comprehensive answers to the first four questions, we completed analyses using dispatch data on call-outs involving all persons aged ≥16 years as well as analyses focusing on only cases involving older persons (defined as ≥65 years). Questions 5 and 6 were addressed using data on call-outs involving all persons aged≥16 years.

## Methods

### Design

A retrospective analysis of dispatch data capturing suspected seizure cases managed by a regional ambulance service during four periods: 1 July 2014 to 31 March 2015 (period I), 1 July 2016 to 31 March 2017 (II), 1 July 2018 to 31 March 2019 (III) and 1 July 2021 to 31 March 2022 (IV). Patients needed to be aged≥16 years and have been alive on ambulance arrival/departure (deaths are uncommon; 0.02%).^19^

The rationale for the periods was as follows. Period I’s start reflects when NWAS’ began coding whether a case’s location was a care home. Period IV’s end is just before NWAS changed its coding system. The 9-month duration of the periods and their spacing is influenced by a preference to have comparable periods while also excluding the coronavirus disease 2019 pandemic.^30^

### Context

#### Service catchment

NWAS is in many respects representative.^22^ Like other English ambulance services, management options available to it are ‘Hear & Treat’ (telephone support only), ‘See & Treat’ (person is attended to by ambulance personnel but stays in place) and ‘See & Convey’—be it to an ED (‘See & Convey to ED’) or an alternative healthcare facility (‘See & Convey Elsewhere’). The latter can include conveyance to both a low-acuity facility (eg, general practitioner or nurse-led urgent care walk-in centres) or high-acuity facility (eg, a specialist obstetric ward, mental health unit).^31^

Box 1 details NWAS’ catchment area. Briefly, it is an area of ~5400 square miles,^32^ encompassing around 1800 care homes; 33% have registered nursing provision.^11^ The proportion of the catchment residing in a care home was stable across the periods (0.87% in period IV) (table 1).^33^ During period IV, the regional prevalence of diagnosed dementia and epilepsy in those aged ≥18 was 0.74% (nationally 0.71%) and 0.93% (nationally 0.80%) respectively.^34^

Box 1 **Description of North West Ambulance Service (NWAS) region**

**NWAS’s regional catchment area in England**

**Key information about NWAS’ catchment:**

- North West Ambulance Service NHS Trust (NWAS) serves the north-west region of England.
- Its catchment area is ~5400 square miles^32^ and includes both remote-rural and urban environments. It covers Merseyside, Greater Manchester, Cheshire, Lancashire and Cumbria.
- The area includes ~1800 care homes, with 33% having registered nursing provision.^11^
- Between period I and IV, the catchment’s population aged ≥16 years increased by 2.2% (from 5 631 981 to 5 756 361). The proportion of the population aged ≥65 years rose between period I and IV by 5.87% (from 22.3% to 23.6%).^56 57^
- In period I, 0.88%^58^ of the catchment’s population aged ≥16 resided in a care home, compared with 0.87% in period IV.^33^ In period I, the proportion of catchment aged ≥65 years residing in a care home was 3.29% compared with 3.01% in period IV.
- The north-west region features high rates of deprivation.^59^ It also has comparatively poorer health. During period IV, the age-standardised percentage of people in the national census reporting they were ‘disabled and limited a lot’ was 9.1% (compared with national level of 7.5%); 6.4% described their general health as ‘bad or very bad’ (compared with a national level of 5.3%).^60^
- During period IV, the regional prevalence of diagnosed dementia and epilepsy in those aged ≥18 was 0.74% (nationally 0.71%) and 0.93% (nationally 0.80%), respectively.^34^
- NWAS, like other ambulance services publish the number of cases related to all presentations that it manages. During period IV, the data show NWAS managed 8 26 873 cases; 3.3% lower than in period III, the earliest period for which comparable data are available.^61^

**Table 1:** Incidence rates within NWAS’ region by time period for care home and wider community separately

	Period	Case location					
		Care home			Wider community		
		Cases*†	Population at risk	Incidence rate(cases per 1000 people/ year)‡(95% CI)	Cases*†	Population at risk	Incidence rate(cases per 1000 people/ year)‡(95% CI)
Cases in those aged≥16	Period I(1 July 2014 to 31 March 2015)	1730	50 109	46.03(43.86 to 48.20)	20 385	5 581 872	4.87(4.80 to 4.94)
	Period II(1 July 2016 to 31 March 2017)	1883	56 612	44.35(42.35 to 46.35)	20 520	5 628 767	4.86(4.79 to 4.93)
	Period III(1 July 2018 to 31 March 2019)	2019	55 363	48.62(46.50 to 50.75)	24 564	5 657 051	5.79(5.72 to 5.86)
	Period IV(1 July 2021 to 31 March 2022)	2095	50 138	55.71(53.33 to 58.10)	25 556	5 706 223	5.97(5.90 to 6.04)
Cases in those aged≥65	Period I(1 July 2014 to 31 March 2015)	1226	41 327	39.55(37.34 to 41.77)	2668	1 217 564	2.92(2.81 to 3.03)
	Period II(1 July 2016 to 31 March 2017)	1280	46 690	36.55(34.55 to 38.56)	2748	1 252 323	2.93(2.82 to 3.04)
	Period III(1 July 2018 to 31 March 2019)	1400	45 321	41.19(39.03 to 43.35)	3616	1 285 209	3.75(3.63 to 3.87)
	Period IV(1 July 2021 to 31 March 2022)	1456	41 044	47.30(44.87 to 49.73)	4011	1 318 181	4.06(3.93 to 4.18)

* A case represents a unique incident for which the ambulance service’s help was sought for and to which it responded (be it by ‘Hear & Treat’, ‘See & Treat’ or ‘See & Convey’).The incidence rates ( cases per people/ year) do not account for some individuals potentially being responsible for during a period. The pseudo-anonymised nature of the data meant we could determine which individuals were responsible for which case.

† A case represents a unique incident for which the ambulance services help was sought for and to which it responded (be it by HearTreat, SeeTreat or SeeConvey);Multiple calls may be made to the ambulance service for the same case (eg, by different members of the public witnessing the same suspected seizure). We accounted for this by removing duplicate calls within the dispatch data for cases with the same location and approximate time.

‡ The incidence rates (ie, cases per 1000 people/ year) do not account for some individuals potentially being responsible for≥1 case during a period. The pseudo-anonymised nature of the data meant we could determine which individuals were responsible for which case.Multiple calls may be made to the ambulance service for the same case ( by different members of the public witnessing the same suspected seizure). We accounted for this by removing duplicate calls within the dispatch data for cases with the same location and approximate time;

NWASNorth West Ambulance Service

#### Coding of ambulance cases

NWAS used the protocol-driven Advanced Medical Priority Dispatch System (AMPDS) to triage and code calls.^35^ AMPDS prompts non-clinical call handlers to ask scripted questions. Cases indicating a suspected seizure are meant to be processed and coded as ‘Protocol 12’. When evaluated against the judgement of attending paramedics, AMPDS is reasonably accurate (sensitivity 71.4%; specificity 99.0%).^19^

According to ‘Protocol 12’, suspected seizure cases are, on the basis of the caller’s answers, classified into one of four subcodes to indicate perceived nature/severity (A(lpha), B(ravo), C(harlie) and D(elta)). A and B are meant to capture lower acuity calls and include scenarios such as ‘Not seizing now and breathing regularly’ (A) and ‘Fitting with effective breathing <35 years'’(B). Subcodes C and D are meant to capture cases more likely to require advanced assessment and/or intervention. C includes instances such as ‘Focal fit (not alert)’ and D includes ‘Not breathing (after key questioning)’ and ‘Continuous or multiple fitting’ (D). US evidence indicates seizure terminating medication is more commonly administered to subcode D cases.^36^

An e-suffix is appended to a case’s subcode if the caller responds in an affirmative way to ‘Is s/he an epileptic? (diagnosed with a fitting disorder)’.

Since late 2017,^37^ NWAS, like all regional ambulance services, also classified cases (including suspected seizures) based on responses to *pretriage* questions into Ambulance Response Programme (ARP) dispatch priority category: 1 (‘life-threatening’), 2 (‘emergency’), 3 (‘urgent’) or 4 (‘non-urgent’). A person described as ‘fitting’, being unconscious, or experiencing breathing difficulties automatically results in category 1 assignment. ARP category determines the urgency with which an ambulance attends. To meet the faster target for higher priority calls, ARP also influences how many resources are initially dispatched.

### Data

For each case, NWAS provided a pseudo-anonymised data extract, including as applicable (depending on the case’s location and period of occurrence) date, time, patient age (restricted to whether they were <or ≥65 to maintain anonymity), AMPDS subcode, ARP, category, location (full postcode and care home coding by NWAS), ambulances dispatched, management, time until arrival and duration. It was supplemented with data to clarify the case’s geographical location (classified according to ‘Sustainability and Transformation Partnership’^38^) and the characteristics of the care homes that cases occurred in. Online supplemental table 1 offers variable details.

For a case to be considered as occurring at a care home, it needed to: (1) be coded as occurring at one by NWAS, with confirmation that the case’s postcode was associated with a care home via the regulator’s register for the period; or (2) occur at a postcode associated with a care home according to the regulator’s register at the time and have involved someone aged ≥65 years. Online supplemental material 2 provides additional detail.

### Analysis

#### Inclusion

For inclusion in analyses, a case needed, as a minimum, data on whether an ambulance was dispatched for it. The proportion with and without this was calculated.

#### Description

Number of cases overall and during each period were determined, along with the proportion from care homes. Side-by-side tables compared care home cases with wider community ones. The proportion of care homes from the catchment with cases occurring at them during each period was determined. Rates at which cases occurred during the periods inside and outside of care homes were calculated and expressed as per 1000 person/years, along with 95% CIs (online supplemental material 3). Rate changes are presented as percentages. All statistics were calculated when including cases involving persons aged ≥16 as well as only when including cases involving persons aged≥65 years.

#### Direct costs

Direct costs were calculated using data for cases involving persons aged ≥16 years. The direct NHS cost associated with an ambulance case depends on how and when it was managed. ‘Hear & Treat’, ‘See & Treat’ and ‘See & Convey’ have different tariffs and these change. We thus, using data on cases involving persons aged ≥16, summed the number of cases managed in each way for each 9-month period, multiplied it by the corresponding tariff, and combined outputs. As case numbers did not fluctuate by calendar month, we also estimated annualised costs. Similar procedures were followed for wider community cases. Online supplemental material 4 gives additional methodological details.

#### Factors associated with management

This analysis used data from period IV and included cases involving persons aged ≥16 years. It examined which available case characteristics were associated with receipt of ‘See & Convey to ED’ management among those (n=27 412 cases) receiving a face-to-face response. Period IV was used as it is most reflective of current ambulance practice. We chose not to restrict age, to allow testing for the influence of the widest range of case characteristics.

As variables capturing case characteristics differed according to whether it occurred in a care home or not (online supplemental table 1), separate analyses were conducted. Univariate analyses (χ^2^ and Mann-Whitney’s U tests) were first used for each model. Variables showing significant associations (α=0.05) with receipt of ‘See & Convey to ED’ were subjected to multivariable binomial logistic regression testing, employing a forward step process. A p value <0.05 was deemed statistically significant.

Analyses were undertaken using Statistical Package for the Social Sciences statistics for Windows, V.29 (Armonk, New York, IBM Corporation).

### Governance

This retrospective analysis of anonymised data was classified as service evaluation rather than research according to the NHS’ Health Research Authority definition and peer-reviewed and approved as such by NWAS (Ref: NWAS_EVAL_0080). This is because it sought to broadly describe service provision, not to change treatment, generate immediately transferable findings, nor judge care against some standard. Ensuring data protection and maintaining patient anonymity were crucial, with access to the data being strictly controlled.

Reporting has been completed according to the Strengthening the Reporting of Observational Studies in Epidemiology. statement for cross-sectional studies.

### Patient and public involvement

The National Institute for Health Research Applied Research Collaborative North West Coast provides the infrastructure by which the public can advise on areas of perceived research priority. The study reported arose following three public advisors sharing personal experiences of relatives residing in care homes who had experienced suspected seizures and then identifying potential opportunities for care improvement. Following four workshops with the advisors and other stakeholders (geriatricians, paramedics, neurology, home managers, general practice, emergency medicine, nurse specialists), an absence of evidence on the topic was confirmed and the study reported here designed. Public advisors helped refine the study design, actively participate in its completion and interpretation. One is a coauthor on this article.

## Results

### Call-outs

#### Number and contribution of care homes

**Figure 1:**
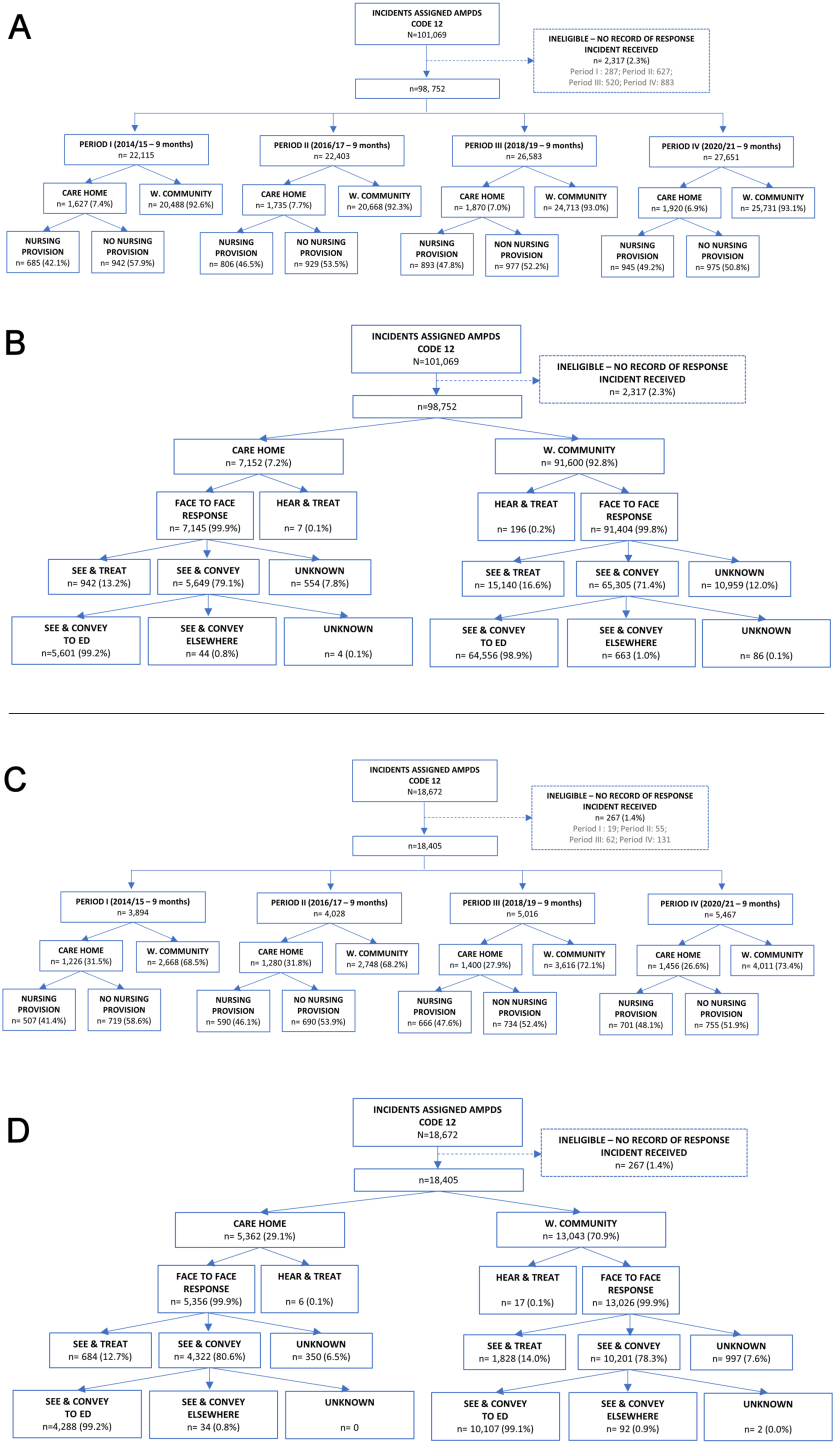
Flow of cases by period and location from which they arose and their management. (A) and (B) relate to cases involving persons aged≥16 years. (C) and (D) relate to cases involving persons aged≥65. Period I=1 July 2014 to 31 March 2015; Period II=1 July 2016 to 31 March 2017; Period III=1 July 2018 to 31 March 2019, Period IV=1 July 2021 to 31 March 2022. No nursing provision, means no registered nursing provision at site according to Care Quality Commission register for time period. ED, emergency department; N/n, number; NWAS, North West Ambulance Service; w. community, wider community.

A total of N=98 752 eligible cases involving persons aged ≥16 occurred across the periods (figure 1A). Of these, 7152 (7.24%, CI 7.08 to 7.40) arose from care homes (range across periods, 6.9% to 7.7%); 3329 (46.5%, CI 45.3 to 47.7) occurred at homes with nursing provision (figure 1B).

Of all cases, n=18 405 (18.6%, CI 18.3 to 18.8) involved persons aged ≥65 years (figure 1C). Of them, 5362 (29.1%, CI 28.4 to 29.7) occurred at a care home. Most (74.9%, CI 73.9 to 75.9) care home cases involved persons aged ≥65 years; 14.2% (CI 14.0 to 14.4) of wider community cases did (table 2).

**Table 2:** Characteristics of suspected seizure cases for whole period when combined and for care home and wider community separately

Periods combined	Age≥16			Age≥65 only		
	Location			Location		
	Anyn (%)	Care homen (%)	Wider communityn (%)	Anyn (%)	Care homen (%)	Wider communityn (%)
Total eligible casesn (%)	98 752 (100)	7152 (7.2)Nursing 3329 (46.5)Non-nursing 3823 (53.5)	91 600 (92.8)	18 405 (18.6)	5362 (29.1)Nursing 2464 (46.0)Non-nursing 2898 (54.0)	13 043 (70.9)
Age (years)						
<65	78 632 (79.6)	1767 (24.7)	76 865 (83.9)	0	0	0
≥65	18 405 (18.6)	5362 (75.0)	13 043 (14.2)	18 405 (18.6)	5362 (100)	13 043 (100)
Unknown	1715 (1.7)	23 (0.3)	1692 (1.8)	0	0	0
AMPDS 12 subcode*						
D range	59 381 (60.1)	4259 (59.5)	55 122 (60.2)	10 045 (54.6)	3012 (56.2)	7033 (53.9)
C range	22 362 (22.6)	2115 (29.6)	20 247 (22.1)	6514 (35.4)	1819 (33.9)	4695 (36.0)
B range	4396 (4.5)	113 (1.6)	4283 (4.7)	134 (0.7)	50 (0.9)	84 (0.6)
A range	12 236 (12.4)	650 (9.1)	11 586 (12.6)	1669 (9.1)	470 (8.8)	1199 (9.2)
Missing	377 (0.4)	15 (0.2)	362 (0.4)	43 (0.2)	11 (0.2)	32 (0.2)
ARP category (period III and IV only)†						
Category 1	32 862 (60.6)	2015 (53.2)	30 847 (61.2)	5365 (51.2)	1419 (49.7)	3946 (51.7)
Category 2	16 223 (29.9)	1512 (39.9)	14 711 (29.2)	4617 (44.0)	1283 (44.9)	3334 (43.7)
Category 3	4838 (8.9)	248 (6.5)	4590 (9.1)	440 (4.2)	141 (4.9)	299 (3.9)
Category 4	305 (0.6)	15 (0.4)	290 (0.6)	60 (0.6)	13 (0.4)	47 (0.6)
Missing	6 (0.0)	0	0	1 (0.0)	0	1 (0.0)
Suffix-e‡						
Yes	39 490 (40.0)	2833 (39.6)	36 657 (40.0)	5275 (28.7)	1642 (30.6)	3.633 (27.9)
Missing	377 (0.4)	15 (0.2)	362 (0.4)	43 (0.2)	11 (0.2)	32 (0.2)
Mode of response						
Face to face	98 549 (99.8)	7145 (99.9)	91 404 (99.8)	18 382 (99.9)	5356 (99.9)	13 026 (99.9)
Telephone alone (Hear & Treat)	203 (0.2)	7 (0.1)	196 (0.2)	23 (0.1)	6 (0.1)	17 (0.1)
Missing	0	0	0	0	0	0
Management						
‘Hear & Treat’	203 (0.2)	7 (0.1)	196 (0.2)	23 (0.1)	6 (0.1)	17 (0.01)
‘See & Treat’	16 082 (16.3)	942 (13.2)	15 140 (16.5)	2512 (13.6)	684 (12.8)	1828 (14.0)
‘See & Convey’	70 954 (71.9)	5649 (79.0)	65 305 (71.3)	14 523 (78.9)	4322 (80.6)	10 201 (78.2)
Missing	11 513 (11.7)	554 (7.7)	10 959 (12.0)	1347 (7.3)	350 (6.5)	997 (7.6)
If conveyed, where to						
‘See & Convey to ED’	70 157 (98.9)	5601 (99.2)	64 556 (98.9)	14 395 (99.1)	4288 (99.2)	10 107 (99.1)
‘See & Convey Elsewhere’	707 (1.0)	44 (0.8)	663 (1.0)	126 (0.9)	34 (0.8)	92 (0.9)
Missing	90 (0.1)	4 (0.1)	86 (0.1)	2 (0.0)	0	2 (0.0)
If face to face response, time until on scene (min)						
Median (IQR)	9 (6–15)	9 (6–16)	9 (6–15)	10 (6–17)	9 (6–16)	10 (6–17)
Missing	112 (0.1)	5 (0.0)	107 (0.1)	0	0	0
If face to face response, number of resources attending						
Median (IQR)	1 (1–2)	1 (1–2)	1 (1–2)	1 (1–2)	1 (1–2)	1 (1–2)
1	59 347 (60.3)	4322 (60.5)	55 025 (60.3)	11 184 (60.8)	3273 (61.1)	7911 (60.7)
2	36 625 (37.2)	2651 (37.1)	33 974 (37.2)	6755 (36.7)	1961 (36.6)	4794 (36.8)
3	2229 (2.3)	157 (2.2)	2072 (2.3)	409 (2.2)	112 (2.1)	297 (2.3)
4	191 (0.2)	9 (0.1)	182 (0.2)	27 (0.1)	9 (0.2)	18 (0.1)
5–8	47 (0.0)	1 (0.0)	46 (0.1)	6 (0.0)	1 (0.0)	5 (0.0)
Missing	110 (0.1)	5 (0.1)	105 (0.1)	1 (0.0)	0	1 (0.0)

* AMPDS 12 subcode A includes situations such as ‘Impending Fit (Aura)’, B ‘Fitting with Effective Breathing<35 years’, C ' ‘Focal fit (not alert),’ and D includes ‘Not breathing (after key questioning)’ or ‘Continuous or multiple fitting’ (D).

† Following the ‘Ambulance Response Programme’^37^ services introduced standardised pre-triage questions, with a view to better targeting resources according to need. Calls are categorised as category 1 (‘life-threatening’, 7-minute min mean response time target from call connect to arrival of first ambulance resource), 2 (‘emergency’, respond 18 minutes min on average), 3 (‘urgent’, respond to 90% in 120 minutes min) or category 4 (‘non-urgent’, respond to 90% in 180 minutes min). A person described at the time of the call as 'fitting', being unconscious, or experiencing breathing difficulties should automatically results in category 1.

‡ The scripted AMPDS question underpinning this is ‘Is s/he an epileptic? (diagnosed with a fitting disorder)’". So-called ‘person first language’ is largely preferred over approaches like this that label a person by their diagnosis.^62^

AMPDS, Advanced Medical Priority Dispatch System; ARP, Ambulance Response Priority; ED, emergency department; nnumber

During the 9-month periods, the proportion of care homes in the catchment within ≥1 call-out involving a person aged ≥65 ranged from 34.7% (period III, median 1, IQR 1–2) to 36.6% (period II; median 1, IQR 1–2) (online supplemental table 2).

#### Incidence rate and change across periods

The incidence rate for care homes was higher than for the wider community for every period, both when considering cases involving persons aged ≥16 and only cases involving persons aged ≥65 (table 1). For instance, for period IV, when considering only cases involving people aged ≥65, incidence was 47.30 (CI 44.87 to 49.73) per 1000 person/year compared with 4.06 (CI 3.93, 4.18).

Incidence in both the care home and the wider community increased between period I and IV. When looking at cases involving those aged ≥16, it increased by 21.0% and 22.6%, respectively.

### Characteristics

#### Nature/severity of cases

The AMPDS profile of care homes cases was broadly similar to that of the wider community. Most were assigned subcode D (table 2), which includes ‘Not breathing…’ and ‘Continuous or multiple fitting’.

A subgroup comparison showed that of care home cases, those in homes with nursing provision rather than without, were more likely to receive subcode D (online supplemental table 3). When considering cases involving those aged ≥65, it was 63.5% (CI 61.6 to 0.65.4) compared with 49.8% (CI 48.0 to 51.7).

#### Reported epilepsy history

A similar proportion of care home and wider community cases was assigned suffix-e to indicate an epilepsy history (table 2). Of cases in care homes involving those aged ≥65, 30.6% (CI 29.3 to 31.8) had a suffix-e.

#### Dispatch priority

Care home cases were somewhat less frequently assigned the highest ARP priority category of 1 (table 2); 49.6% (CI 47.8 to 51.5) of care home cases involving persons aged ≥65 had this category compared with 51.7% (CI 50.6 to 52.8) of wider community cases.

Of care home cases, those occurring in homes with, rather than without, nursing provision were more often assigned category 1 (online supplemental table 3).

#### Timing

Care home cases exhibited similar occurrence rates across days of the week and calendar months within the time periods. This also applied to wider community cases.

The diurnal variation in call volume for care home cases differed from the wider community (online supplemental figure 1). When considering cases involving persons aged ≥65 years, within care homes, there was a rapid rise in calls from a nadir of 3:00, peaking at 8:00–9:00, followed by a rapid decline to the nadir; 32.0% (CI 30.8 to 33.3) of the cases occurred between the nadir and 9:00. In the wider community, there was in contrast an increase in call volume from a nadir of 4:00 to a peak between 10:00 and 12:00, gradually decreasing back to the nadir.

### Management

#### Mode of response and speed

Almost all care home and wider community cases received a face-to-face response (figure 1B,D). Median time between ‘call pick-up’ and scene arrival was also comparable (table 2). When considering cases in care homes involving those aged ≥65, it was 9 min (IQR 6–16).

#### Response approach and time on scene

Care home cases that were attended to were more often managed by ‘See & Convey to ED’ than wider community cases (figure 1B). Of cases involving persons aged ≥65, 80.0% (CI 78.9 to 81.1) of care home cases were conveyed to ED, compared with 77.5% (CI 76.8 to 78.3) of wider community cases (figure 1D).

Time ‘on scene’ for care home and wider community cases resulting in ‘See & Convey’ was similar. For cases involving those aged ≥65, it was 37 min (IQR 29 to 48) for care home cases and 37 min (29 to 49) for wider community ones.

Comparing all periods to identify any change in case management was not appropriate due to missing data on exact management for 25.2% of cases in period I and 26.5% of cases in period II. A comparison of management in periods III and IV was possible and indicated a slight reduction in use of ‘See & Convey to ED’. When considering cases involving persons aged ≥65, it reduced from 79.4% (CI 77.2 to 81.5) to 71.3% (CI 68.9 to 73.6) for care homes cases and from 76.6% (CI 75.2 to 78.0) to 73.5% (CI 72.1 to 74.9) for wider community cases.

### Costs

The total direct cost to the ambulance service of managing all the cases during the periods was £24 205 033. The combined annualised total for the four periods was £32 273 377. The mean cost associated with cases occurring inside and outside of care homes during the respective periods was similar (eg, £329.57 vs £325.47 during period IV). Consequently, care homes accounted for a proportionate 7.26% of the direct costs (£1 756 570) (online supplemental material 4).

### Factors associated with conveyance to ED

#### Care home cases

Among the 1915 period IV care home cases involving persons age ≥16, 70.4% resulted in ‘See & Convey to ED’. Univariate analyses identified nine variables as significantly associated with management (online supplemental table 4). As there was a strong correlation between ARP category and AMPDS subcode, only ARP was entered into the model.

In the adjusted model, the following were, in descending order of importance, significantly associated with lower likelihood of receiving a ‘See & Convey to ED’ response rather than ‘See & Treat’/‘See & Convey Elsewhere’: occurrence in the Lancashire and South Cumbria area rather than Greater Manchester Health and Social Care Partnership (reference), occurrence in a specialist learning disability (LD) home, lower ARP priority code, having one rather than two ambulances dispatched and non-occurrence within a home with nursing provision (table 3).

**Table 3:** Multivariable models of association between case characteristics and management according to location

Variable		Location					
		Care home*			Wider community†		
		Beta coefficient	Adjusted OR95% CI		Beta coefficient	Adjusted OR95% CI	
ARP priority category‡	Category 1	0.277	1.319 (1.054 to 1.650)	p=0.015	0.187	1.206 (1.131 to 1.286)	p<0.001
	Category 2	Reference	1		Reference	1	
	Category 3	−0.460	0.631 (0.413 to 0.969)	p=0.033	−0.545	0.580 (0.515 to 0.653)	p<0.001
Suffix-e§	No	ns	ns	ns	Reference	1	
	Yes				−0. 459	0.632 (0.598 to 0.668)	p<0.001
Patient≥65 years	No	ns	ns	ns	Reference	1	
	Yes				0.305	1.357 (1.242 to 1.458)	p<0.001
Ambulances dispatched	One	Reference	1		Reference	1	
	Two	0.271	1.311 (1.020 to 1.684)	p=0.034	0.406	1.4831 (1.393 to 1.577)	p<0.001
Geographical area (STP)¶	Lancashire and South Cumbria						
	STP	−0.376	0.687 (0.532 to 0.886)	p=0.004	−0.146	0.864 (0.807 to 0.925)	p<0.001
	Cheshire and Merseyside STP	−0.081	0.922 (0.723 to 1.175)	p=0.571	−0.012	0.988 (0.927 to 1.054)	p=0.721
	Greater Manchester HSCP STP	Reference	1		Reference	1	
	Cumbria and Noth East STP	−0.015	0.985 (0.537 to 1.806)	p=0.962	−0.451	0.637 (0.555 to 0.739)	p<0.001
Learning disabilities ‘specialist’ care home	No	Reference	1		–	–	–
	Yes	−0.359	0.649 (0.517 to 0.943)	p=0.019			
Nursing provision at care home	No	Reference	1		–	–	–
	Yes	0.204	1.226 (1.002 to 1.500)	p=0.048			
Day of week	Sat/Sunday	ns	ns	ns	Reference	1	
	Monday–Friday				−0.083	0.921 (0.868 to 0.976)	p=0.006

Reference, several variables had >2 categorical responses. When this was the case, one category was chosen as the reference category and dummy indicator variables were created for entry into the regression models, if one dummy indicator variable was identified as being significantly related to the outcome, all the dummy indicators were retained in the model.

* Negelkerke’s R2=0.037.

† Negelkerke’s R2=0.05.

‡ Following the ‘Ambulance Response Programme’^37^ services introduced standardised pre-triage questions, with a view to better targeting resources according to need. Calls are categorised as category 1 (‘life-threatening’, 7-minute min mean response time target from call connect to arrival of first ambulance resource), 2 (‘emergency’, respond 18 minutes min on average), 3 (‘urgent’, respond to 90% in 120 minutes min) or category 4 (‘non-urgent’, respond to 90% in 180 minutes min). A person described at the time of the call as 'fitting', being unconscious, or experiencing breathing difficulties should automatically results in category 1;.

§ The scripted AMPDS question presented within the text underpinning this is ‘Is s/he an epileptic? (diagnosed with a fitting disorder)’". So-called ‘person first language’ is largely preferred over approaches like this that label a person by their diagnosis.^62^;

¶ Cases were, by their postcode, classified according to an aggregated geographic area of relevance for the time periods examined –, namely, Sustainability and Transformation Partnership (STP). STPs comprised local NHS organisations and Local Authorities drawing up shared proposals (‘place-based plans’) to improve health and care in the areas they serve. Integrated care boards succeeded them in July 2022.^63^

AMPDS, Advanced Medical Priority Dispatch System; ARP, Ambulance Response Priority; ns, not statistically significant; STP, sustainability and transformation partnership

#### Wider community

Of the 25 497 period IV cases involving persons’ age ≥16, 67.6% resulted in ‘See & Convey to ED’. Within the adjusted model, the following were significantly associated with a lower likelihood of a ‘See & Convey to ED’ response: having one rather than two ambulances dispatched, lower ARP priority code, presence of a suffix-e, occurrence in the area of Lancashire and South Cumbria or Cumbria and North East rather than Greater Manchester, patient age <65 years, and weekday occurrence (table 3).

## Discussion

### Main findings

Our study, while exploratory, represents a crucial step in understanding seizure-related needs within care homes. Despite accommodating approximately 0.8% of the regional population, care homes accounted for about 7.2% of suspected seizure cases requiring ambulance attention and costs. While the proportion of residents in care homes remained steady, incidence increased by a fifth between 2014 and 2022. This suggests a rising occurrence of seizures and/or epilepsy in care homes and/or a greater propensity to seek assistance. Care home cases were more often conveyed to ED than wider community cases, despite presentations potentially being less severe.

### Practice and policy implications

#### For seizure specialists

The revealed incidence of seizure-related cases in care homes should be noted. The pseudo-anonymised nature of our dataset prevented us from determining how many individuals accounted for cases. However, our findings suggest a considerable number of residents necessitate specialist support. The high call-out incidence rate within the care home population compared with the wider population might be due to a combination of interacting factors. Namely: conditions that give rise to seizures disproportionately affect older adults^3 4^; recent qualitative work suggests a tendency in some care homes to call ambulances for suspected seizures, even when there may not be a clinical need^39^; and, finally, older adults face barriers in accessing specialist care for seizures, which may result in poorer seizure control and more frequent ambulance callouts. Further research is though, needed before definitive conclusions can be made.

In the immediate term, the priority should be on ensuring specialist services have the capacity to meet the demand that appears to exist within care homes and that care pathways are organised to facilitate referrals to them. Our dataset was ‘unlinked’. Thus, we do not know how many cases were being supported by seizure specialists. A next step would be for specialist services to audit requests received for support for individuals from care homes and consider whether under-referral is occurring and why. Reuber *et al*.’s^40^ framework might help.

One factor that did *not* emerge as associated with non-conveyance in our model for care homes was a report of an epilepsy history (suffix-e). We expected it to be; ED conveyance is *not* mandated for those with an established diagnosis.^23^ A suffix-e *was* significant in the community model—reducing likelihood of conveyance by 36%. Perhaps paramedics are less confident in, and supported by, reports of epilepsy histories for care home residents. Older people are more often started on anti-seizure medication by non-seizure specialists. The UK’s National Audits of Seizure Management in Hospitals found people aged≥60 were twice as likely to have a history of seizures and be on medication, but not have a diagnosis and were less likely to have seen a specialist or be referred to one.^41^ Diagnoses could thus be less secure and unlikely to be accompanied by meaningful care plans to direct seizure management. This highlights one consequence of inequitable referrals.

#### For ambulance services

A care home is ostensibly a favourable place to have and recover from an uncomplicated seizure. Paramedics should not be affected by factors operating in the wider community which can create momentum for conveyance.^22 42 43^ Our findings revealed that cases from care homes were though, more frequently conveyed than wider community cases. For some other presentations, being in a care home is associated with lower conveyance.^44^ Dispatch data lack the granularity to permit assessment of the appropriateness of the conveyances. Future studies should consider this given older individuals are at increased risk of adverse outcomes from hospital stays.

The poorer health of residents in care homes^6^ might be one reason for their frequent conveyance. It is possible that paramedics encounter more individuals in care homes with abnormal observations or signs deemed worthy of ED consideration. Care home residents might even have different, more severe forms of epilepsy. However, we had limited data on the health of the individuals responsible for the call-outs and so could not adjust for health differences to test such explanations. We were able to only adjust for age. Poorer health on behalf of residents, including dementia, may also complicate paramedics ability to determine whether the person is recovering to baseline. Older persons can experience more prolonged confusion post-seizure.^16^ It is noteworthy though, that paramedics spent similar time on scene for care home and wider community cases before conveying. Paramedics indicate that perceived time pressures contribute to conveyance.^22^ Ambulance services might consider explicitly allowing paramedics more time for care home case assessment.

Another potential reason could be case timing. Care home cases unusually peaked at 8:00/9:00. The reasons for the spike are unclear. Some seizure presentations occur more frequently during or on awakening from sleep.^45^ Care home staff may simply be more engaged with residents in the morning, leading to more seizure recognition. Either way, it may impede paramedics’ ability to access resources and pathways supporting management other than ED, as many operate only during 'core hours'.^46^ Service commissioners should consider this to prevent age discrimination.

#### For care homes

Within our regression analysis, we found home type was associated with whether conveyance to ED occurred. Having accounted for reported severity, occurrence within a home specialising in LDs reduced likelihood of ED conveyance by 35%. This might be attributable to the established association between LDs and epilepsy. Such homes might be better prepared to manage seizures and paramedics and home staff more comfortable with individuals recovering within them. This should be explored. If the suggestion arises that staff in non-specialist homes should receive training similar to that of LD home staff, any intervention should account for potential learning decay, as ambulance cases were frequent for some homes but not for most (40% in each period had at least one case).

One potentially relevant difference between care homes that do and do not specialise in LDs is the prescription of emergency rescue medication to their residents with histories of prolonged or repeated tonic-clonic seizures and the training their staff have in their use. Persons with LDs are at high risk of status epilepticus so rescue prescriptions often considered.^47^ It would be important to understand if the prescription rate for persons with such seizure patterns in non-specialist LD homes is optimal given such treatments could permit care home staff to intervene early^48^ and potentially reduce the need for conveyance to ED, even if an ambulance arrives.

Home type was found to be important in another way: cases within homes with nursing provision had a 23% higher risk of ED conveyance. This may be attributable to the typically poorer health of residents in such homes compared with those without nursing provision.^11^ Nurses, due to their extended training, may also only seek ambulances for more severe cases.

### Strengths and limitations

The study has revealed the role of care homes in ambulance seizure cases. This, and the narrow confidence intervals surrounding its estimates, is a strength. Another is the examination of temporal changes, representing a first for seizure cases inside and outside of care homes.

Regarding potential limitations, our findings are derived from a regional service. Replicating the study elsewhere would help understand generalisability. We also used routine data. It was at times missing and its pseudo-anonymised nature prevented some analyses. We also relied on AMPDS coding. It does capture suspected seizure cases well.^19^ However, there is no evidence from England on the ability of Protocol 12’s subcodes and suffix-e to capture different histories and severities.

Comparing the AMPDS and ARP data for care home and wider community cases has some challenges. While dispatch data suggest care home presentations were somewhat less severe than those in the wider community, we cannot rule out that care home staff waited longer before seeking ambulance support, potentially resulting in patients being in more favourable states when assessed by call operators.

Our study was unable to fully elucidate the factors influencing ED conveyance. Elsewhere we report an effort to engage with care home staff for insights.^39^ Future modelling efforts may though, also consider incorporating distance between the case and ED and the extent of infrastructure in the local region that supports non-conveyance to ED (eg, alternative destinations such as urgent care centres or access to rapid reviews by visiting healthcare professionals for residents with uncomplicated seizures). These factors may explain the geographic variations we found. Access to additional data on cases, such as paramedics' observations, could also help. Future modelling should also seek more granular detail on home ownerships.^49^ With regards the modelling we completed we also highlight that variables were selected for testing based on their significance in univariate tests. This approach is not without potential limitations. There was though, limited empirical or theoretical evidence to guide more informed selection.

More detailed case data could also offer insights into the aetiology of suspected seizure cases. This might help explain the observed increase in cases which we cannot currently explain. Specifically, the incidence of suspected seizure cases inside and outside of care homes increased by around 20% between the period I and IV. This was despite NWAS’ coding remaining consistent, incidence and prevalence of epilepsy not increasing^50^ and seizure freedom in those diagnosed not deteriorating.^51^ Moreover, the starting incidence within the region was not unusually low.^18^ Previous examinations of ambulance data for other presentations in the UK and beyond have reported year-on-year increases in call numbers that cannot be satisfactorily explained by population growth.^52 53^ Factors such as an increasing proportion of older adults within the population, deteriorating access to alternative services^54^ and/or a lowering of the threshold for calling an ambulance have been hypothesised as possible explanations.^55^

## Conclusions

Care homes account for a disproportionate amount of suspected seizure cases requiring ambulance attention and incidence could be increasing. Nearly all cases are conveyed to EDs and factors beyond reported severity may influence this. Work is needed to check residents can and are receiving equitable seizure specialist input into their care and to understand the clinical necessity of the conveyances to ED.

## supplementary material

10.1136/bmjopen-2024-089126 online supplemental file 1

10.1136/bmjopen-2024-089126 online supplemental file 2

10.1136/bmjopen-2024-089126 online supplemental file 3

10.1136/bmjopen-2024-089126 online supplemental file 4

10.1136/bmjopen-2024-089126 online supplemental file 5

10.1136/bmjopen-2024-089126 online supplemental file 6

10.1136/bmjopen-2024-089126 online supplemental file 7

10.1136/bmjopen-2024-089126 online supplemental file 8

10.1136/bmjopen-2024-089126 online supplemental file 9

## Data Availability

Data are available upon reasonable request.
