# Supplementary material for: ‘Blue-lighting’ seizure-related needs in care homes: a retrospective analysis of ambulance call-outs for seizures in North West England (2014–2021), their management and costs, with community comparisons
Source: BMJ Open. 2024 Nov 13;14(11):e089126. doi: 10.1136/bmjopen-2024-089126 (PMC11574507; doi:10.1136/bmjopen-2024-089126)
Supplement: online supplemental file 1 [file bmjopen-14-11-s001.docx]

**Supplementary material 1** Systematic literature search: Methods and summary of studies

**Scope**

A systematic literature search was conducted to identify existing evidence on the seizure-related emergency care needs of persons within care home settings in high income countries whose health care system is principally publicly funded.

**Methods**

*Identification*

Searches of titles were made using Medline and Scopus from inception until 08/12/2023. Search terms were informed by Davies et al.’s strategy and ^1^ including the following (with adaptations for different databases):

1. care home OR care homes OR nursing home OR nursing homes OR nursing care OR  nursing facilit* OR residential home OR  residential  OR  resident*  OR  institutional care  OR  respite care  OR  nightingale home  OR  nightingale homes
2. AND  epilep*  OR  seizure  OR seizures OR  convuls*

*Eligibility and screening*

To be eligible an article had to be reported in English, be a primary study (reviews and commentaries and perspectives were ineligible), published in a peer-reviewed journal, have been published on or after 1^st^ January 2000, and have considered seizure-related emergency care needs in the care home residents (studies of persons who had visited and stayed in assessment units for diagnostic purposes were ineligible). Given the specified scope, only studies completed in Europe, Australia, New Zealand and Canada could also be included. The reasons for this were that:

1. Epilepsy’s burden, including seizure freedom, varies between countries, with the burden of active epilepsy inversely correlating with a country’s developmental status.^2^
2. Incidence and prevalence of epilepsy in older adults varies around the world, with a prevalence peak in older adults having not been found by some studies in parts of Asia, India, Latin America and the Caribbean. ^3^ Some of these regions are obviously too large and diverse to be considered as a whole. However, given the scope of the search task, a decision was made to exclude them.
3. The United States was excluded since it is the only Organization for Economic Cooperation and Development country whose health care system is not principally p public funded ^4^ and because cost barriers to medical treatment there may result in an unusual pattern of emergency care use.^5^

Titles/abstracts for identified articles were screened for eligibility by two reviewers (SL & AN), removing duplicates and obviously irrelevant studies. Agreement between them was high and all discrepancies were resolved through discussion, without the need for a third reviewer. Full texts versions of the articles that they both agreed as ostensibly eligible were accessed.

**Results**

The selection process and reasons for exclusion are shown in the PRISMA flow chart below (Figure S1.1. Only 1 eligible study, by Magnusson et al. was identified. It is summarised in Table S1.1.

**PRISMA Flow chart showing identification and selection assessment**

Scopus

(n=138)

Medline

(n = 113)

## Identification

Unique articles identified (n = 251)

Articles after duplicates removed & screened

(n = 211)

## Eligibility

Excluded after title and abstract review
(n =189)

Articles reviewed

(n = 22)

Excluded after full article review

(n =21)

Eligible articles identified

(n =1)

## Included

**Summary of findings from eligible studies identified by systematic search**

| **Title** | **Lead Author** | **Year** | **Country** | **Objective/s** | **Study years/ Population size** | **Findings of relevance to scope** |
| --- | --- | --- | --- | --- | --- | --- |
| High-resolution mapping of epilepsy prevalence, ambulance use, and socioeconomic deprivation in an urban area of Sweden | Magnusson C. | 2019 | Sweden | Compare 1) epilepsy prevalence and 2) emergency medical service cases for seizures in areas of different socioeconomic standing in the urban area of Gothenburg. | 2013-18 for objective 2  ~690,000 permanent inhabitants in Gothenburg. | - Within a local population with a reported epilepsy prevalence of 0.51%, 7,907 ambulance cases for suspected seizures were recorded between 2013 and 2018. - Of these cases, 901 (11.4%) occurred at “residential homes”. The remainder occurred within the wider community. - Correlation analysis showed significant but weak associations between the number of ambulance cases per capita in each district and proportion of welfare recipients (r = .31, P < .0001), income (r = −.19, P < .0001) and a negligible, albeit significant, association with age (r = −.13, P = .0125). - Proportion ambulance cases transported to hospital overall was 83.7%. It was not reported for locations separately, nor by time period. |

**REFERENCES**

1. Davies SL, Goodman C, Bunn F, et al. A systematic review of integrated working between care homes and health care services. *BMC Health Services Research* 2011;11:320.

2. GBD 2016 Epilepsy Collaborators. Global, regional, and national burden of epilepsy, 1990-2016: a systematic analysis for the Global Burden of Disease Study 2016. *The Lancet Neurology* 2019;18(4):357–75.

3. Beghi E, Giussani G, Costa C, et al. The epidemiology of epilepsy in older adults: A narrative review by the ILAE Task Force on Epilepsy in the Elderly. *Epilepsia* 2023;64(3):586-601.

4. Colombo F, Tapay N. Private Health Insurance in OECD Countries: The Benefits and Costs for Individuals and Health Systems: Paris; 2004 [Available from: <https://www.oecd.org/els/health-systems/33698043.pdf> accessed 12th March 2024.

5. Docteur E, Suppanz H, Woo J. The US health system: an assessment and prospective directions for reform Paris2003 [Available from: <https://one.oecd.org/document/ECO/WKP(2003)4/en/pdf> accessed 12th March 2024.
