# Supplementary material for: ‘Blue-lighting’ seizure-related needs in care homes: a retrospective analysis of ambulance call-outs for seizures in North West England (2014–2021), their management and costs, with community comparisons
Source: BMJ Open. 2024 Nov 13;14(11):e089126. doi: 10.1136/bmjopen-2024-089126 (PMC11574507; doi:10.1136/bmjopen-2024-089126)
Supplement: online supplemental file 2 [file bmjopen-14-11-s002.docx]

**SUPPLEMENTARY MATERIAL 2** Categorisation of cases according to whether they occurred at a care home or not

From 2014, the North-West Ambulance Service’s (NWAS) computer aided dispatch (CAD) system logged whether case location was considered to be a care home. The basis for this classification was an NWAS database that primarily resulted from a one-off exercise which involved NWAS identifying care home addresses in their catchment. Its historical nature meant it had potentially reduced validity to categorise the location of cases in more recent time periods. Hence, a case was categorised as having arisen at a care home if either of the following situations applied:

1. The case was flagged by NWAS as being a care home case and the Care Quality Commission’s (CQC) official register for the time period identified the case’s postcode as being a care home. Most (92.1%) cases involving individuals aged ≥16 flagged as having arisen at a care home by NWAS were confirmed to have done so when checked against the CQC register.
2. Postcodes for all cases involving individuals aged ≥65 year that had not been identified by NWAS as having arisen from a care home were cross-referenced with the CQC’s care home register for the time period. Cases at locations with postcodes matching one on the register were categorized as originating from a care home. An additional n=512 (7.2%) cases across the four time periods involving individuals aged ≥16 were categorised as occurring at a care home via this process. Postcodes in England are specific (averaging 15 households); hence the likelihood of false classification was minimal.

Please note that, like others (e.g.,^1^), we assumed persons attended to at care home locations were care home residents (rather than visitors), and that patients treated at other locations were not.

**REFERENCES**

1. Sinclair DR, Charlton K, Stow D, et al. Care Home Residency and Its Association with Ambulance Service Workload. *Journal of the American Medical Directors Association* 2023;24(5):657–60.
