# Supplementary material for: ‘Blue-lighting’ seizure-related needs in care homes: a retrospective analysis of ambulance call-outs for seizures in North West England (2014–2021), their management and costs, with community comparisons
Source: BMJ Open. 2024 Nov 13;14(11):e089126. doi: 10.1136/bmjopen-2024-089126 (PMC11574507; doi:10.1136/bmjopen-2024-089126)
Supplement: online supplemental file 3 [file bmjopen-14-11-s003.docx]

**Supplementary MATERIAL 3** Details of methods used to calculate incidence rates

Incidence rates were calculated for ambulance cases across the whole population for each time period and also for the care home and wider populations separately. This was done using cases involving persons aged ≥16 and also for just cases involving persons aged ≥65. The approach used is explained below.

***Method to calculate incidence rate for whole catchment for each period***

The rate at which ambulance cases occurred are expressed as per 1,000 person-years. To do this, for each time period, incidence per people-months was first calculated (cases during period/ [number of people aged ≥16 or ≥65 as appropriate within NWAS’ catchment* 9 months of period]). This was then multiplied by 12 to create an incidence per people-year and multiplied by 1,000. Confidence intervals were calculated using StatsDirect Version 3.0.171.

To permit these calculations it was necessary to define the size of the population aged ≥16 and ≥65 years within NWAS’ catchment area during each time period. Thus, NWAS detailed its catchment and Lower Layer Super Output Areas (LSOA) for it were extracted. Office for National Statistics (ONS) small area population data was used to identify the number of people aged ≥16 and ≥65 living within these LSOAs during the different time periods.

***Methods used to calculate separate incidence rates for populations residing inside and outside of care homes***

Using a similar formula to that stated above, separate incidence rates were calculated for each time period for the care home population and for the wider community. This required estimates of the number people within the catchment area aged ≥16 and ≥65 that were and were not living in a care home during the different periods. These were calculated as follows:

*To estimate those within the catchment area aged ≥16 that were and were not living in a care home during the different periods:*

For each period, the Care Quality Commission’s (CQC) register of care homes was accessed and their postcodes converted into LSOAs. Any with a LSOA within NWAS’ catchment area for the period was identified.

Having excluded homes identified by the CQC as being specifically registered to care for children, we then, as per Wolters et al. ^1^ and Hancock et al.,^2^ extracted information on how many beds the CQC register recorded these homes had. The number of beds was totalled and multiplied by a factor indicating the expected bed occupancy rate for care homes in the UK for the time period (range 79.5 to 88.9%).^3^

The resulting figures were taken to represent the number of people living within care homes during the period. It was deducted from the total number of people aged ≥16 years estimated to reside within the catchment for that period to estimate the number of people within the time period living within the wider community (Table below).

*To estimate those within the catchment area aged ≥65 that were and were not living in a care home during the different periods:*

The same steps were followed as above with the exception that the number of people estimated to be living within care homes based needed to be refined to identify the number that were aged ≥65 years. To do this, we multiplied the number estimated to be living in care homes in the different periods by a factor provided the English and Welsh decennial census which describes the proportion of the care residents in England that are aged ≥65. A factor of .8247 from the 2011 Census ^4^ was used for Periods I and II and a factor of .8185 from the 2021 Census ^5^ was used for Periods III and IV (Table below).

**Estimated number of people within NWAS catchment area residing inside and outside care home for the different periods**

| **PERIOD** | **Population within NWAS catchment area ^a^** | | | | **Care home facilities within NWAS catchment area ^b^** | | | | | **Wider community residents within nwas catchment area** | | |
| --- | --- | --- | --- | --- | --- | --- | --- | --- | --- | --- | --- | --- |
|  | ***All ages*** | ***≥16 years*** | ***>65 years*** | ***Number of registered care homes*** | | ***Number of registered homes excluding those for children*** | ***Number of people aged ≥16 years anticipated to be within care homes excluding those for children:***  *(Number of beds*occupancy rate for period) ^c^* | ***Number of people aged ≥65 years anticipated to be within care homes excluding those for children***  *(Number of people aged ≥16 years within care home** *factor on age profile of residents). ^d^* | ***Number of people aged ≥16 years AND expected to be residing outside of care home setting*** | | ***Number of people aged ≥65 years AND expected to be residing outside of care home setting*** |  |
| ***Period I***  (1/7/2014 to 31/3/2015 | 6,939,566 | 5,631,981 | 1,258,891 | 2,109 | | 1,969 | 50,109 | 41,327 | 5,581,872 | | 1,217,564 |  |
| ***Period II***  (1/7/2016 to 31/3/2017) | 7,014,367 | 5,685,379 | 1,299,013 | 2,035 | | 2,005 | 56,612 | 46,690 | 5,628,767 | | 1,252,323 |  |
| ***Period III***  (1/7/2018 to 31/3/2019) | 7,064,980 | 5,712,414 | 1,330,530 | 1,962 | | 1,933 | 55,363 | 45,321 | 5,657,051 | | 1,285,209 |  |
| ***Period IV***  (1/7/2021 to 31/3/2022) | 7,120,758 | 5,756,361 | 1,359,225 | 1,926 | | 1,886 | 50,138 | 41,044 | 5,706,223 | | 1,318,181 |  |

***Notes:*** NWAS, North-West Ambulance Service National Health Service Trust

^a^ To define the size of the population, Office for National Statistics (ONS) estimates for the middle period of the calendar year that formed most of the time period was used. Mid-2014 for Period I, ^6^ mid-2016 for Period II,^7^ and mid-2018 for Period III.^8^ For Period IV data, mid-2021 was not available at the time of analysis and its release date was unclear. Thus, the nearest available was used – namely, mid-2020. ^9^

^b^ As the Care Quality Commission (CQC) regularly updates its register throughout a calendar year, the register produced closest to the mid-point of each of the time periods was used to quantify the number of registered care homes within NWAS’ catchment area, who they cater for and their bed capacity – namely, November 2014 for Period I,^10^ November 2016 for Period II,^11^ November 2018 for Period III,^12^ and November 2021 for Period IV. ^13^

^c^ Occupancy rates for the different periods were sourced those published by Statista for different periods for the UK.^3^

^d^ The factor was derived from English and Welsh decennial census> it which describes the proportion of the care residents in England that are aged ≥65. A factor of .8247 from the 2011 census was used for Periods I and II and a factor of .8185 from the 2021 Census was used for Periods III and IV.

**REFERENCES**

1. Wolters A, Santos F, Lloyd T, et al. Emergency admissions to hospital from care homes: how often and what for? 2019 [Available from: <https://www.health.org.uk/publications/reports/emergency-admissions-to-hospital-from-care-homes> accessed 11th March 2024.

2. Hancock J, Matthews J, Ukoumunne OC, et al. Variation in ambulance call rates for care homes in Torbay, UK. *Health and Social Care in the Community* 2017;25(3):932–37.

3. Statista. Care home occupancy rate in the United Kingdom (UK), from 2006 to 2021 2021 [Available from: <https://www.statista.com/statistics/1231777/care-home-occupancy-in-the-uk/> accessed 11th March 2024.

4. Office for National Statistics. Communal establishment management and type by sex by age 2013 [Available from: <https://www.nomisweb.co.uk/census/2011/DC4210EWLA/view/2092957703?rows=c_cectmcews11&cols=position_age> accessed 10 May 2024.

5. Office for National Statistics. Communal establishment and household population characteristics, England and Wales: Census 2021 2023 [Available from: <https://www.ons.gov.uk/peoplepopulationandcommunity/housing/datasets/communalestablishmentandhouseholdpopulationcharacteristicsenglandandwalescensus2021> accessed 10 May 2024.

6. Office for National Statistics. Table SAPE20DT1: Mid-2014 Population Estimates for Lower Layer Super Output Areas in England and Wales by Single Year of Age and Sex - Supporting Information 2018 [Available from: <https://www.ons.gov.uk/file?uri=/peoplepopulationandcommunity/populationandmigration/populationestimates/datasets/lowersuperoutputareamidyearpopulationestimates/mid2014/sape20dt1mid2014lsoasyoaestimatesformatted.zip> accessed 6th March 2024.

7. Office for National Statistics. Table SAPE20DT1: Mid-2016 Population Estimates for Lower Layer Super Output Areas in England and Wales by Single Year of Age and Sex - Supporting Information 2018 [Available from: <https://www.ons.gov.uk/file?uri=/peoplepopulationandcommunity/populationandmigration/populationestimates/datasets/lowersuperoutputareamidyearpopulationestimates/mid2016/sape20dt1mid2016lsoasyoaestimatesformatted.zip> accessed 11th March 2024.

8. Office for National Statistics. Table SAPE21DT1a: Mid-2018 Population Estimates for Lower Layer Super Output Areas in England and Wales by Single Year of Age and Sex - Supporting Information 2019 [Available from: <https://www.ons.gov.uk/file?uri=/peoplepopulationandcommunity/populationandmigration/populationestimates/datasets/lowersuperoutputareamidyearpopulationestimates/mid2018sape21dt1a/sape21dt1amid2018on2019lalsoasyoaestimatesformatted.zip> accessed 11th March 2024.

9. Office for National Statistics. Table SAPE23DT2: Mid-2020 Population Estimates for Lower Layer Super Output Areas in England and Wales by Single Year of Age and Sex - Supporting Information 2021 [Available from: <https://www.ons.gov.uk/file?uri=/peoplepopulationandcommunity/populationandmigration/populationestimates/datasets/lowersuperoutputareamidyearpopulationestimates/mid2020sape23dt2/sape23dt2mid2020lsoasyoaestimatesunformatted.xlsx> accessed 6th March 2024.

10. Care Quality Commission. 01 November 2014 HSCA Active locations for providers registered under the Health and Social Care Act 2014 [Available from: <https://docs.google.com/spreadsheets/d/1O64bbqM1gPlctGmaXXx31RGWEMLvgVEh/edit#gid=2079504308> accessed 6th March 2024.

11. Care Quality Commission. 01 November 2016 HSCA Active locations for providers registered under the Health and Social Care Act 2016 [Available from: <https://docs.google.com/spreadsheets/d/1ui7ioW521bthgR-BHQmImJbYsUt6nX3I/edit#gid=2079807962> accessed 11th March 2024.

12. Care Quality Commission. 01 November 2018 HSCA Active locations for providers registered under the Health and Social Care Act 2018 [Available from: <https://docs.google.com/spreadsheets/d/1DZ3zooCgl5ybHEFXydsj8id8iJcQxnds/edit#gid=1625369923> accessed 11th March 2024.

13. Care Quality Commission. 01 November 2021 HSCA Active locations for providers registered under the Health and Social Care Act 2021 [Available from: <https://drive.google.com/file/d/1D_m7-khNdu-wmJnhIEUn-SczglwYhfL9/view?usp=drive_link> accessed 6th March 2024.
