# Supplementary material for: ‘Blue-lighting’ seizure-related needs in care homes: a retrospective analysis of ambulance call-outs for seizures in North West England (2014–2021), their management and costs, with community comparisons
Source: BMJ Open. 2024 Nov 13;14(11):e089126. doi: 10.1136/bmjopen-2024-089126 (PMC11574507; doi:10.1136/bmjopen-2024-089126)
Supplement: online supplemental file 4 [file bmjopen-14-11-s004.docx]

**SUPPLEMENTARY MATERIAL 4** Direct cost tariffs and cost calculations

**Tariff charged by ambulance service for different management response according to period**

| **Management approach** | **PERIOD** | | | |
| --- | --- | --- | --- | --- |
|  | **Period I** | **Period II** | **Period III** | **Period IV** |
| ***‘Hear & treat’*** | £65.34 | £27.25 | £26.20 | £103.59 |
| ***‘See & treat’*** | £129.87 | £144.94 | £183.78 | £251.03 |
| ***‘See & Convey’*** | £212.54 | £211.96 | £229.30 | £362.04 |

***Notes:*** Tariff information provided by R. Marsden, Head of Finance, NWAS (personal communication, 23.6.2023); Tariff is applied regardless of the code or pretriage code assigned to case. There is also no difference in tariff charged for cases that result in conveyance to an ED (i.e., ‘See & Convey to ED) or conveyance elsewhere (i.e., ’See & Convey elsewhere’); Period I, 1/7/2014 to 31/3/2015; Period II, 1/7/2016 to 31/3/2017; Period III, 1/7/2018 to 31/3/2019, Period IV, 1/7/2021 to 31/3/2022.

**Direct cost to the ambulance service arising from cases according to period and location**

| **PERIOD** | **All** | **location** | |
| --- | --- | --- | --- |
|  |  | ***Care home*** | ***Wider community*** |
| ***Period I*** | | | |
| Cases | 22,115 | 1627 | 20,488 |
| For whom costs extrapolated ^a^ | 5,581 | 246 | 5,335 |
| Total | £4,700,322.10 | £345,802.58 | £4,354,519.52 |
| Typical cost | £212.54 | £212.54 | £212.54 |
| Annualised | £6,267,096.13 | £461,070.11 | £5,806,026.03 |
|  |  |  |  |
| ***Period II*** | | | |
| Cases | 22,403 | 1,735 | 20,668 |
| For whom costs extrapolated ^a^ | 5,931 | 308 | 5,623 |
| Total | £4,748,539.88 | £367,750.60 | £4,380,789.28 |
| Typical cost | £211.96 | £211.96 | £211.96 |
| Annualised | £6,331,386.51 | £490,334.13 | £5,841,052.37 |
|  |  |  |  |
| ***Period III*** | | | |
| Cases | 26,583 | 1,870 | 24,713 |
| For whom costs extrapolated ^a^ | 0 | 0 | 0 |
| Total | £5,748,769.56 | £410,250.30 | £5,338,519.26 |
| Typical cost | £216.26 | £219.39 | £216.02 |
| Annualised | £7,665,026.08 | £547,000.40 | £7,118,025.68 |
|  |  |  |  |
| ***Period IV*** | | | |
| Cases | 27,651 | 1,920 | 25,731 |
| For whom costs extrapolated ^a^ | 1 | 0 | 1 |
| Total | £9,007,401.80 | £632,767.33 | £8,374,634.19 |
| Typical cost | £325.75 | £329.57 | £325.47 |
| Annualised | £12,009,869.07 | £843,689.77 | £11,166,178.92 |

***Notes***: ^a^ For a minority of cases (11.7% in total), how they were managed (beyond it being noted that an ambulance was dispatched) was missing. Therefore, as per Dickson et al.’s ^1^ method, costs for them were estimated by extrapolating it from the cases without missing data during the period; Period I, 1/7/2014 to 31/3/2015; Period II, 1/7/2016 to 31/3/2017; Period III, 1/7/2018 to 31/3/2019, Period IV, 1/7/2021 to 31/3/2022.

**REFERENCES**

1. Dickson JM, Taylor LH, Shewan J, et al. Cross-sectional study of the prehospital management of adult patients with a suspected seizure (EPIC1). *BMJ Open* 2016;6:e010573.
