# Supplementary material for: ‘Blue-lighting’ seizure-related needs in care homes: a retrospective analysis of ambulance call-outs for seizures in North West England (2014–2021), their management and costs, with community comparisons
Source: BMJ Open. 2024 Nov 13;14(11):e089126. doi: 10.1136/bmjopen-2024-089126 (PMC11574507; doi:10.1136/bmjopen-2024-089126)
Supplement: online supplemental file 5 [file bmjopen-14-11-s005.docx]

**Supplementary TABLE 1** Information obtained, as applicable, for each suspected seizure case according to period and their data source

| **Data item** | **Periods recorded for** | **Details** | **Coding and type** | **Case locations available for** | | **Source** |
| --- | --- | --- | --- | --- | --- | --- |
|  |  |  |  | ***Care***  ***home*** | ***Wider community*** |  |
| ***Age*** | *All* | Was the patient recorded as being aged ≥65 years at the time | Nominal  (Yes, No, Age unknown/ not recorded by ambulance service) | Yes | Yes | CAD |
| ***Hour of day*** | *All* | Time call received | Q/continuous (0-24) | Yes | Yes | CAD |
| ***Day of week*** | *All* | Day of week call received | Nominal (Mon-Sun) | Yes | Yes | CAD |
| ***Month*** | *All* | Month call received | Nominal  (1-12) | Yes | Yes | CAD |
| ***Response priority*** | *Period III, IV* | ARP priority category ^a^ | Ordinal  (1-5; 1 ‘life-threatening; 2 ‘emergency’, 3 ‘urgent’ 4, ‘non-urgent’) | Yes | Yes | CAD |
| ***Nature*** | *All* | APMDS 12 subcode ^b^ | Ordinal  (A range, B range, C range, D range, Uknown) | Yes | Yes | CAD |
| ***Suffix-e*** | *All* | “Is s/he an epileptic? (diagnosed with a fitting disorder )” ^c^ | Nominal  (Yes, No, Unknown) | Yes | Yes | CAD |
| ***Number ambulances dispatched*** | *All* | Total number of ambulance resources attending case  (if an ambulance was dispatched) | Continuous | Yes | Yes | CAD |
| ***Geographic location*** | *Period IV* | Sustainability and Transformation Partnership (STP) region ^d^ | Nominal  (Lancashire & South Cumbria STP; Cheshire & Merseyside STP; Greater Manchester HSCP STP; Cumbria & Noth East STP) | Yes | Yes | CAD |
| ***Deprivation*** | *Period IV* | IMD decile for case postcode location ^e^ | Ordinal | Yes | Yes | CAD |
| ***Time until on scene*** | *All* | Minutes between call and first ambulance resource arriving on scene  (if an ambulance was dispatched) | Continuous | Yes | Yes | CAD |
| ***Management response*** | *All* | Broad categorisation of management | Nominal  (‘Hear & Treat’, ‘See & Treat’, ‘See & Convey’) | Yes | Yes | CAD |
| ***Time on scene*** | *All (only conveyed cases)* | How long did the paramedics spend on scene (calculated as time between first resources arrival on scene and last resource to leave) | Continuous | Yes | Yes | CAD |
| ***Conveyance destination*** | *All (categorised as ‘See & Convey’)* | Broad categorisation of type of facility person was conveyed to when ‘See & Convey’ was recorded as management response. ^f^ | Nominal (‘See & Conveyed to ED’; ‘See & Convey Elsewhere’) | Yes | Yes | CAD |
| ***Occurrence at care home*** | *All* | Whether case occurred at a care home  (see Supplementary File 3 for criteria) | Binary  (Yes, No) | Yes | Yes | CAD |
| ***If care home*** |  |  |  |  |  |  |
| ***Nursing provision*** | *All* | Any registered nursing provision on care home site | Binary  (Yes, No) | Yes | No | CQC |
| ***Size of home*** | *Period IV* | Number of beds in care home | Continuous | Yes | No | CQC |
| ***Ownership type*** | *Period IV* | Home’s ownership type | Nominal (Individual; NHS body; organisation; partnership) | Yes | No | CQC |
| ***Charity involvement*** | *Period IV* | Home’s charity status | Binary  (Yes a charity, Not a charity) | Yes | No | CQC |
| ***Quality rating*** | *Period IV* | Latest overall care quality rating for home ^g^ | Ordinal  (No CQC rating available; Inadequate, Requires Improvement, Good, Outstanding) | Yes | No | CQC |
| ***Was quality rating inherited?*** | *Period IV* | Was the care home’s quality rating inherited from a previous owner of the home? | Binary  (Not applicable, Yes, No) | Yes | No | CQC |
| ***Dementia specialist home?*** | *Period IV* | Does the care home state it is a specialist provider for persons with dementia? ^h^ | Binary  (No, not a specialist; Yes, a specialist) | Yes | No | CQC |
| ***Learning disability or autism specialist home?*** | Period IV | Does the care home state it is a specialist provider for persons with learning disability? ^h^ | Binary  (No, not a specialist; Yes, a specialist) | Yes | No | CQC |
| ***Epilepsy specialist provider?*** | Period IV | Is the care home a specialist epilepsy provider? ^h^ | Binary  (No, not a specialist; Yes, a specialist) | Yes | No | Local knowledge exercise |

***Notes****:* AMPDS, Advanced Medical Priority Dispatch System; ARP, Ambulance Response Priority; CAD, Computer Aided Dispatch system; CQC, Care Quality Commission; IMD, Index of Multiple Deprivation; STP, Sustainability and Transformation Partnership; The pseudo-anonymised data extract from the North-West Ambulance Service for each case was supplemented with data ^1-5^ to clarify the case's geographical area (classified according to the 'Sustainability and Transformation Partnership'[42] occurring in) and characteristics of any care homes cases occurred in.

^a^ Following the ‘Ambulance Response Programme’ ^6^ services introduced standardised pre-triage questions, with a view to better targeting resources according to need. Calls are categorised as category 1 (‘life-threatening’, 7-minute mean response time target from call connect to arrival of first ambulance resource), 2 (‘emergency’, respond 18 minutes on average), 3 (‘urgent’, respond to 90% in 120 minutes) or category 4 (‘non-urgent’, respond to 90% in 180 minutes). A person described at the time of the call as 'fitting', being unconscious, or experiencing breathing difficulties should automatically results in category 1;

^b^ AMPDS 12 subcode A includes situations such as 'Impending Fit (Aura)', B 'Fitting with Effective Breathing<35 years', C ' Focal fit (not alert)’, and D includes 'Not breathing (after key questioning)' or 'Continuous or multiple fitting' (D);

^c^ Whilst the scripted AMPDS question is "Is s/he an epileptic? (diagnosed with a fitting disorder)", so-called ‘person first language’ is largely preferred over approaches like this that label a person by their diagnosis.^7^;

^d^ Cases were, using their postcode, classified according to an aggregated geographic area of relevance for the time periods examined – namely, Sustainability and Transformation Partnership (STP). STPs comprised local NHS organisations and Local Authorities drawing up shared proposals (‘place-based plans’) to improve health and care in the areas they serve. Integrated care boards succeeded them in July 2022.^8^

^e^ For each case, an area-based measure of social deprivation was obtained to indicate the relative social deprivation of the postcode location for the case. It was obtained by linking the case’s postcode with its English Index of Multiple Deprivation (IMD) 2019 ^9^ score and classifying it by deprivation decile (1 being the most social deprived; 10 least being the least deprived).

^f^ Any case recorded as having been taken to a hospital ED was classified as having received a ‘See & Convey to ED’ response. Cases recorded as having been conveyed to a designation other than an ED were classified as having received a ‘See & Convey Elsewhere’ response. For 31% of ‘See & Convey’ cases, a hospital was named as the destination for conveyance, but the specific department was missing. We thus cross-referenced the hospital site to central NHS data ^10^ to determine the facilities offered. If it included a Type 1 ED, the case was classified as ‘See and Convey to ED’; if it did not it was classified as ‘See and Convey Elsewhere’.

^g^ During the periods the regulator assessed homes according to a 5 domains ^11^: Safety, Effective, Caring, Responsive, and Well-led. An overall quality rating can be generated with scoring options including, Outstanding (“service is performing exceptionally well”), Good (“service is performing well and meeting our expectations”), Requires improvement (“service is not performing as well as it should and we have told the service how it must improve”) and Inadequate (“service is performing badly and we've taken action against the person or organisation that runs it”).

^h^ Regulations require that care homes describe the range of peoples’ needs their home intends to meet. Homes must state any special ‘service user bands’ they intend to provide for. Whether the home has the specific knowledge, skills, training or facilities to do this is part of how is assess the home. Dementia and learning disability are amongst the service user bands that homes can state. There is no service user band for epilepsy. Therefore, we established a list of homes known in the region to provide for epilepsy on the basis of knowledge of a regional neurologists, epilepsy nurses, researchers and a charity that operate a national epilepsy service advice line (Epilepsy Action).

**REFERENCES**

1. Care Quality Commission. 01 November 2014 HSCA Active locations for providers registered under the Health and Social Care Act 2014 [Available from: <https://docs.google.com/spreadsheets/d/1O64bbqM1gPlctGmaXXx31RGWEMLvgVEh/edit#gid=2079504308> accessed 6th March 2024.

2. Office for National Statistics. LSOA (2011) to Clinical Commissioning Groups to Sustainability and Transformation Partnerships (April 2020) Lookup in England 2024 [Available from: <https://www.data.gov.uk/dataset/d64dd9bd-acfc-431f-98ef-c4f002248b05/lsoa-2011-to-clinical-commissioning-groups-to-sustainability-and-transformation-partnerships-april-2020-lookup-in-england> accessed 6th March 2024.

3. Care Quality Commission. 01 November 2016 HSCA Active locations for providers registered under the Health and Social Care Act 2016 [Available from: <https://docs.google.com/spreadsheets/d/1ui7ioW521bthgR-BHQmImJbYsUt6nX3I/edit#gid=2079807962> accessed 6th March 2024.

4. Care Quality Commission. 01 November 2018 HSCA Active locations for providers registered under the Health and Social Care Act 2018 [Available from: <https://docs.google.com/spreadsheets/d/1DZ3zooCgl5ybHEFXydsj8id8iJcQxnds/edit#gid=1625369923> accessed 6th March 2024.

5. Care Quality Commission. 01 November 2021 HSCA Active locations for providers registered under the Health and Social Care Act 2021 [Available from: <https://drive.google.com/file/d/1D_m7-khNdu-wmJnhIEUn-SczglwYhfL9/view?usp=drive_link> accessed 6th March 2024.

6. NHS England. Ambulance Response Programme 2024 [Available from: <https://www.england.nhs.uk/urgent-emergency-care/improving-ambulance-services/arp/> accessed 6th March 2024.

7. Noble AJ, Robinson A, Snape D, et al. 'Epileptic', 'epileptic person' or 'person with epilepsy'? Bringing quantitative and qualitative evidence on the views of UK patients and carers to the terminology debate. *Epilepsy & Behavior* 2017;67:20-27.

8. NHS England. ODS Implementation of Integrated Care Boards from July 2022 2022 [Available from: <https://digital.nhs.uk/services/organisation-data-service/upcoming-code-changes/implementation-of-icbs-from-april-2022> accessed 11th March 2024.

9. Ministry of Housing CLG. English indices of deprivation 2019 2019 [Available from: <https://www.gov.uk/government/statistics/english-indices-of-deprivation-2019> accessed 18 March 2024.

10. NHS. Find a hospital 2024 [Available from: <https://www.nhs.uk/service-search/hospital> accessed 6th March 2024.

11. Care Quality Commission. The five key questions we ask 2022 [Available from: <https://www.cqc.org.uk/about-us/how-we-do-our-job/five-key-questions-we-ask> accessed 11th March 2024.
