# Supplementary material for: ‘Blue-lighting’ seizure-related needs in care homes: a retrospective analysis of ambulance call-outs for seizures in North West England (2014–2021), their management and costs, with community comparisons
Source: BMJ Open. 2024 Nov 13;14(11):e089126. doi: 10.1136/bmjopen-2024-089126 (PMC11574507; doi:10.1136/bmjopen-2024-089126)
Supplement: online supplemental file 6 [file bmjopen-14-11-s006.docx]

**SUPPLEMENTARY TABLE 2** Number and proportion of care homes within ambulance service’s catchment area that had a seizure ambulance case occur at them during the different periods and number

| **AGE** | **PERIOD** | **Number of registered homes in region (excluding those for children)** ^a^ | **Care homes**  **with ≥1 case** | **Proportion of homes**  **in region** | **Number of cases at homes with ≥1 case** | | | |
| --- | --- | --- | --- | --- | --- | --- | --- | --- |
|  |  |  |  |  | ***Median*** | ***IQR*** | ***Min*** | ***Max*** |
| ***All incidents in those aged ≥16*** | ***Period I*** | 1969 | 794 | 40.3 | 1 | 1-3 | 1 | 15 |
|  | ***Period II*** | 2005 | 792 | 39.5 | 2 | 1-3 | 1 | 31 |
|  | ***Period III*** | 1933 | 844 | 43.6 | 2 | 1-3 | 1 | 17 |
|  | ***Period IV*** | 1886 | 820 | 43.5 | 2 | 1-3 | 1 | 14 |
| ***Restricted to those aged ≥65*** | ***Period I*** | 1969 | 721 | 36.6 | 1 | 1-2 | 1 | 9 |
|  | ***Period II*** | 2005 | 722 | 36.0 | 1 | 1-2 | 1 | 15 |
|  | ***Period III*** | 1933 | 671 | 34.7 | 1 | 1-2 | 1 | 12 |
|  | ***Period IV*** | 1886 | 664 | 35.2 | 1 | 1-2 | 1 | 11 |

***Notes:*** IQR, interquartile range; Min, minimum; Max, maximum.

^a^ Number of registered homes based on the Care Quality Commission (CQC) register. As it regularly updates its register throughout a calendar year, the register produced closest to the mid-point of each of the time periods was used – namely, November 2014 for Period I,^1^ November 2016 for Period II,^2^ November 2018 for Period III,^3^ and November 2021 for Period IV. ^4^ Period I, 1/7/2014 to 31/3/2015; Period II, 1/7/2016 to 31/3/2017; Period III, 1/7/2018 to 31/3/2019, Period IV, 1/7/2021 to 31/3/2022.

**REFERENCES**

1. Care Quality Commission. 01 November 2014 HSCA Active locations for providers registered under the Health and Social Care Act 2014 [Available from: <https://docs.google.com/spreadsheets/d/1O64bbqM1gPlctGmaXXx31RGWEMLvgVEh/edit#gid=2079504308> accessed 6th March 2024.

2. Care Quality Commission. 01 November 2016 HSCA Active locations for providers registered under the Health and Social Care Act 2016 [Available from: <https://docs.google.com/spreadsheets/d/1ui7ioW521bthgR-BHQmImJbYsUt6nX3I/edit#gid=2079807962> accessed 11th March 2024.

3. Care Quality Commission. 01 November 2018 HSCA Active locations for providers registered under the Health and Social Care Act 2018 [Available from: <https://docs.google.com/spreadsheets/d/1DZ3zooCgl5ybHEFXydsj8id8iJcQxnds/edit#gid=1625369923> accessed 11th March 2024.

4. Care Quality Commission. 01 November 2021 HSCA Active locations for providers registered under the Health and Social Care Act 2021 [Available from: <https://drive.google.com/file/d/1D_m7-khNdu-wmJnhIEUn-SczglwYhfL9/view?usp=drive_link> accessed 6th March 2024.
