# Supplementary material for: ‘Blue-lighting’ seizure-related needs in care homes: a retrospective analysis of ambulance call-outs for seizures in North West England (2014–2021), their management and costs, with community comparisons
Source: BMJ Open. 2024 Nov 13;14(11):e089126. doi: 10.1136/bmjopen-2024-089126 (PMC11574507; doi:10.1136/bmjopen-2024-089126)
Supplement: online supplemental file 7 [file bmjopen-14-11-s007.docx]

**SUPPLEMENTARY TABLE 3** Comparison of the AMPDS 12 subcode and ARP categories of cases within care homes with and without nursing provision

| **PERIODS COMBINED** | **CARE HOME cases** | | |
| --- | --- | --- | --- |
|  | ***Combined*** | ***With nursing provision*** | ***No nursing provision*** |
| ***Total eligible cases***  n (%) | 7,152 | 3,329 | 3,823 |
| ***AMPDS 12 subcode*** ^a^  D range  C range  B range  A range  Missing | 4,259 (59.5)  2,115 (29.6)  113 (1.6)  650 (9.1)  15 (0.2) | 2,214 (66.5)  866 (26.0)  39 (1.2)  206 (6.2)  4 (0.1) | 2,045 (53.5)  1,249 (32.7)  74 (1.9)  444 (11.6)  11 (0.3) |
| ***ARP category (Period III and IV only)*** ^b^  Category 1  Category 2  Category 3  Category 4  Missing | 2,015 (53.2)  1,512 (39.9)  248 (6.5)  15 (0.4) | 1,085 (59.0)  643 (35.0)  102 (5.5)  6 (0.3)  2 (0.1) | 930 (47.6)  869 (44.5)  146 (7.5)  7 (0.4)  3 (0.2) |
| ***Suffix-e*** ^c^  Yes  Missing | 2,833 (39.6)  15 (0.2) | 1387 (41.7)  4 (0.1) | 1446 (37.8)  11 (0.3) |

***Notes:*** AMPDS, Advanced Medical Priority Dispatch System; ARP, Ambulance Response Priority.

^a^ AMPDS 12 subcode A includes situations such as 'Impending Fit (Aura)', B 'Fitting with Effective Breathing<35 years', C ' Focal fit (not alert)', and D includes 'Not breathing (after key questioning)' or 'Continuous or multiple fitting' (D); ^b^ Following the ‘Ambulance Response Programme’ ^1^ services introduced standardised pre-triage questions, with a view to better targeting resources according to need. Calls are categorised as category 1 (‘life-threatening’, 7-minute mean response time target from call connect to arrival of first ambulance resource), 2 (‘emergency’, respond 18 minutes on average), 3 (‘urgent’, respond to 90% in 120 minutes) or category 4 (‘non-urgent’, respond to 90% in 180 minutes). A person described at the time of the call as 'fitting', being unconscious, or experiencing breathing difficulties should automatically results in category 1; ^c^ The scripted AMPDS question underpinning this is "Is s/he an epileptic? (diagnosed with a fitting disorder)". So-called ‘person first language’ is largely preferred over approaches like this that label a person by their diagnosis.^2^ Period I, 1/7/2014 to 31/3/2015; Period II, 1/7/2016 to 31/3/2017; Period III, 1/7/2018 to 31/3/2019, Period IV, 1/7/2021 to 31/3/2022.

**REFERENCES**

1. NHS England. Ambulance Response Programme 2024 [Available from: <https://www.england.nhs.uk/urgent-emergency-care/improving-ambulance-services/arp/> accessed 6th March 2024.

2. Noble AJ, Robinson A, Snape D, et al. 'Epileptic', 'epileptic person' or 'person with epilepsy'? Bringing quantitative and qualitative evidence on the views of UK patients and carers to the terminology debate. *Epilepsy & Behavior* 2017;67:20-27.
