# Supplementary material for: ‘Blue-lighting’ seizure-related needs in care homes: a retrospective analysis of ambulance call-outs for seizures in North West England (2014–2021), their management and costs, with community comparisons
Source: BMJ Open. 2024 Nov 13;14(11):e089126. doi: 10.1136/bmjopen-2024-089126 (PMC11574507; doi:10.1136/bmjopen-2024-089126)
Supplement: online supplemental file 8 [file bmjopen-14-11-s008.docx]

**SUPPLEMENTARY TABLE 4** Unadjusted associated between case characteristics and management

| **Variable** | | | **LOCATION** | | | | | |
| --- | --- | --- | --- | --- | --- | --- | --- | --- |
|  |  |  | ***Care Home*** | | | ***Wider Community*** | | |
|  |  |  | *‘See & convey to ED’* | *‘See & treat’/ ‘See & convey elsewhere’* | *Statistical significance* | *‘See & convey to ED’* | *‘See & treat’/ ‘See & convey elsewhere’* | *Statistical significance* |
| ***Data source*** | N |  | 1,349 | 566 |  | 17,234 | 8,263 |  |
| *NWAS CAD* | ARP priority category, N(%)^a^ | Category 1  Category 2  Category 3 | 798 (59.2)  492 (36.5)  57 (4.2) | 271 (47.9)  250 (44.2)  45 (8.0) | P < 0.001^1^ | 11,802 (68.5)  4,733 (27.5)  681 (4.0) | 5,042 (61.0)  2,410 (29.2)  781 (9.5) | P < 0.001^1^ |
|  | AMPDS 12 subcode, N(%) | 12As/Bs  12Cs  12Ds | 67 (4.9)  496 (38.6)  779 (57.7) | 48 (8.5)  252 (44.5)  264 (46.6) | P < 0.001^1^ | 999 (5.8)  4,858 (28.2)  11,232 (65.2) | 1,054 (12.8)  2,367 (28.6)  4,773 (57.8) | P < 0.001^1^ |
|  | Suffix-e, N (%) ^b^ | No  Yes | 903 (66.9)  439 (32.5) | 352 (62.4)  211 (37.3) | P = 0.13^1^ | 12,308 (71.4)  4,781 (27.7) | 4,898 (59.3)  3,296 (39.9) | P < 0.001^1^ |
|  | Patient ≥65, N (%) | No  Yes | 303 (22.5)  1,039 (77.0) | 147 (26.0)  416 (73.5) | P = 0.54^1^ | 14, 039 (81.5)  2,951 (17.1) | 7, 042 (85.2)  1, 047 (12.7) | P <0.001^1^ |
|  | Hour of day, N(%) | 0 – 5:59  6 – 11:59  12 – 17:59  18 – 23:59 | 146 (10.8)  508 (37.7)  452 (33.5)  243 (18.8) | 54 (9.4)  213 (37.6)  183 (32.3)  116 (20.5) | P = 0.55^1^ | 2,768 (16.1)  3,929 (22.8)  5,570 (32.3)  4,967 (28.8) | 1,300 (15.7)  1,766 (21.4)  2,688 (32.5)  2,509 (30.4) | P = 0.18^1^ |
|  | Day of week, N(%) | Sat/Sunday  Mon - Friday | 376 (29.9)  973 (72.1) | 159 (28.1)  407 (71.9) | P = 0.92^1^ | 5,347 (31.0)  11, 887 (69.0) | 2,400 (29.0)  5,863 (71.0) | P =0.001^1^ |
|  | Month of year, N(%) | Quarter 1  Quarter 3  Quarter 4 | 413 (30.6)  476 (35.3)  460 (34.1) | 179 (31.8)  206 (36.4)  181 (32.0) | P = 0.67^1^ | 5,358 (31.1)  6,201 (36.0)  5,675 (32.9) | 2,527 (30.6)  2,907 (36.3)  2,739 (33.1) | P = 0.71^1^ |
|  | Ambulances dispatched, N (%) | One  Two+ | 930 (68.9)  419 (31.1) | 438 (77.7)  126 (22.3) | P < 0.001^1^ | 10.965 (63.6)  6,269 (36.4) | 6,052 (73.6)  2,173 (26.4) | P < 0.001^2^ |
|  | Minutes until arrival on scene | Median (IQR)  Range | 11 (12)  (0, 816) | 13 (26)  (0, 772) | P < 0.001^2^ | 9 (9)  (0, 899) | 10 (13)  (0, 1,553) | P < 0.001^2^ |
|  | Geographic area (STP), N(%) ^c^ | Lancashire & South Cumbria STP  Cheshire & Merseyside STP  Greater Manchester HSCP STP  Cumbria & Noth East STP | 325 (24.1)  492 (36.5)  489 (36.2)  43 (3.2) | 181 (32.0)  191 (33.7)  178 (31.4)  16 (2.8) | P = 0.005^1^ | 4150 (24.3)  5680 (33.2)  6711 (39.3)  955 (3.2) | 2216 (27.0)  2607 (31.8)  2964 (36.1)  416 (5.1) | P < 0.001^1^ |
|  | Deprivation decile, N(%)^d^ | One  Two  Three  Four  Five  Six  Seven  Eight  Nine  Ten | 275 (20.4)  195 (14.5)  205 (15.3)  151 (11.2)  87 (6.4)  68 (5.0)  94 (7.0)  124 (9.2)  79 (5.9)  70 (5.6) | 90 (15.9)  85 (15.0)  80 (14.1)  69 (12.2)  49 (8.7)  41 (7.2)  39 (6.9)  45 (8.0)  36 (6.4)  32 (5.7) | P = 0.21^1^ | 5,516 (32.3)  2,441 (14.3)  1,648 (9.6)  1,547 (9.0) 1,104 (6.5)  996 (5.8)  842 (4.9)  1, 051 (6.1)  843 (4.9)  1, 108 (6.5) | 2,731 (33.3)  1,225 (14.9)  835 (10.2)  779 (9.5)  535 (6.5)  541 (6.6)  357 (4.4)  385 (4.7)  336 (4.1)  477 (5.8) | P< 0.001^1^ |
| *CQC for care home cases* | Number of beds in care home | Median (IQR)  Range | 44 (30)  (3, 214) | 42.5 (31)  (3, 214) | P =0.016^2^ | - | - | - |
|  | Nursing provision at care home, N (%) | No  Yes | 661 (49.0)  688 (51.0) | 713 (55.3)  253 (44.7) | P = 0.012^1^ | - | - | - |
|  | Care home ownership, N (%) | Individual  NHS  Organisation  Partnership | 28 (2.1)  1 (0.1)  1,289 (95.6)  31 (2.3) | 22 (3.9)  1 (0.2)  527 (93.1)  16 (2.8) | N/A | - | - | - |
|  | Care home charity status, N (%) | No  Yes | 1,228 (91.0)  121 (9.0) | 518 (91.5)  48 (8.5) | P = 0.73^1^ | - | - | - |
|  | Care home quality rating, N (%) ^e^ | None  Good/Outstanding  Inadequate/Requires improvement | 25 (1.9)  986 (73.1)  338 (25.1) | 10 (1.8)  439 (76.6)  117 (20.7) | P = 0.12^1^ | - | - | - |
|  | Inherited rating, N (%) | No  Yes | 1,207 (88.5)  117 (8.7) | 500 (88.3)  56 (9.9) | P=0.69^1^ |  | - | - |
|  | Dementia ‘specialist’ care home, N (%) ^f^ | No  Yes | 386 (28.9)  963 (71.4) | 171 (30.2)  395 (69.8) | P = 0.48^1^ | - | - | - |
|  | Learning disabilities ‘specialist’ care home,  N (%) ^f^ | No  Yes | 1,213 (89.8)  136 (10.1) | 485 (85.7)  81 (14.3) | P = 0.008^1^ | - | - | - |
|  | Epilepsy ‘specialist’ care home, N (%) ^f^ | No  Yes | 1,343 (99.6)  6 (0.4) | 565 (99.8)  1 (0.2) | P = 0.38^1^ | - | - | - |

***Notes****:* AMPDS, Advanced Medical Priority Dispatch System; ARP, Ambulance Response Priority; CAD, Computer Aided Dispatch system; CQC, Care Quality Commission; NWAS, North-West Ambulance Service; STP, Sustainability and Transformation Partnership. ^1^ Chi-squared, ^2^ Mann-Whitney U test. N/A, p-value not computed for this variable due to only 1 observation being present in two of its cells.

^a^  Following the ‘Ambulance Response Programme’ ^1^ services introduced standardised pre-triage questions, with a view to better targeting resources according to need. Calls are categorised as category 1 (‘life-threatening’, 7-minute mean response time target from call connect to arrival of first ambulance resource), 2 (‘emergency’, respond 18 minutes on average), 3 (‘urgent’, respond to 90% in 120 minutes) or category 4 (‘non-urgent’, respond to 90% in 180 minutes). A person described at the time of the call as 'fitting', being unconscious, or experiencing breathing difficulties should automatically results in category 1.

^b^ The scripted AMPDS question presented within the text underpinning this is "Is s/he an epileptic? (diagnosed with a fitting disorder)". So-called ‘person first language’ is largely preferred over approaches like this that label a person by their diagnosis.^2^

^c^ Cases were, by their postcode, classified according to an aggregated geographic area of relevance for the time periods examined – namely, Sustainability and Transformation Partnership (STP). STPs comprised local NHS organisations and Local Authorities drawing up shared proposals (‘place-based plans’) to improve health and care in the areas they serve. Integrated care boards succeeded them in July 2022.^3^

^d^ For each case, an area-based measure of social deprivation was obtained to indicate the relative social deprivation of the postcode location for the case. It was obtained by linking the case’s postcode with its English Index of Multiple Deprivation (IMD) 2019 ^4^ score and classifying it by deprivation decile (1 is the most deprived decile; 10 is the least deprived).

^e^ During the periods the regulator assessed homes according to a 5 domains ^5^: Safety, Effective, Caring, Responsive, and Well-led. An overall quality rating can be generated with scoring options including, Outstanding (“service is performing exceptionally well”), Good (“service is performing well and meeting our expectations”), Requires improvement (“service is not performing as well as it should and we have told the service how it must improve”) and Inadequate (“service is performing badly and we've taken action against the person or organisation that runs it”).

^f^ Regulations require that care homes describe the range of peoples’ needs their home intends to meet. Homes must state any special ‘service user bands’ they intend to provide for. Whether the home has the specific knowledge, skills, training or facilities to do this is part of how is assess the home. Dementia and learning disability are amongst the service user bands that homes can state. There is no service user band for epilepsy. Therefore, we established a list of homes known in the region to provide for epilepsy on the basis of knowledge of a regional neurologists, epilepsy nurses, researchers and a charity that operate a national epilepsy service advice line (Epilepsy Action).

**REFERENCES**

1. NHS England. Ambulance Response Programme 2024 [Available from: <https://www.england.nhs.uk/urgent-emergency-care/improving-ambulance-services/arp/> accessed 6th March 2024.

2. Noble AJ, Robinson A, Snape D, et al. 'Epileptic', 'epileptic person' or 'person with epilepsy'? Bringing quantitative and qualitative evidence on the views of UK patients and carers to the terminology debate. *Epilepsy & Behavior* 2017;67:20-27.

3. NHS England. ODS Implementation of Integrated Care Boards from July 2022 2022 [Available from: <https://digital.nhs.uk/services/organisation-data-service/upcoming-code-changes/implementation-of-icbs-from-april-2022> accessed 11th March 2024.

4. Ministry of Housing CLG. English indices of deprivation 2019 2019 [Available from: <https://www.gov.uk/government/statistics/english-indices-of-deprivation-2019> accessed 18 March 2024.

5. Care Quality Commission. The five key questions we ask 2022 [Available from: <https://www.cqc.org.uk/about-us/how-we-do-our-job/five-key-questions-we-ask> accessed 11th March 2024.
