# Supplementary material for: ‘Blue-lighting’ seizure-related needs in care homes: a retrospective analysis of ambulance call-outs for seizures in North West England (2014–2021), their management and costs, with community comparisons
Source: BMJ Open. 2024 Nov 13;14(11):e089126. doi: 10.1136/bmjopen-2024-089126 (PMC11574507; doi:10.1136/bmjopen-2024-089126)
Supplement: online supplemental file 9 [file bmjopen-14-11-s009.docx]

**SUPPLEMENTARY FIGURE 1** Hour of day at which cases involving persons a) aged ≥16 years and b) ≥65 years occurred according to location


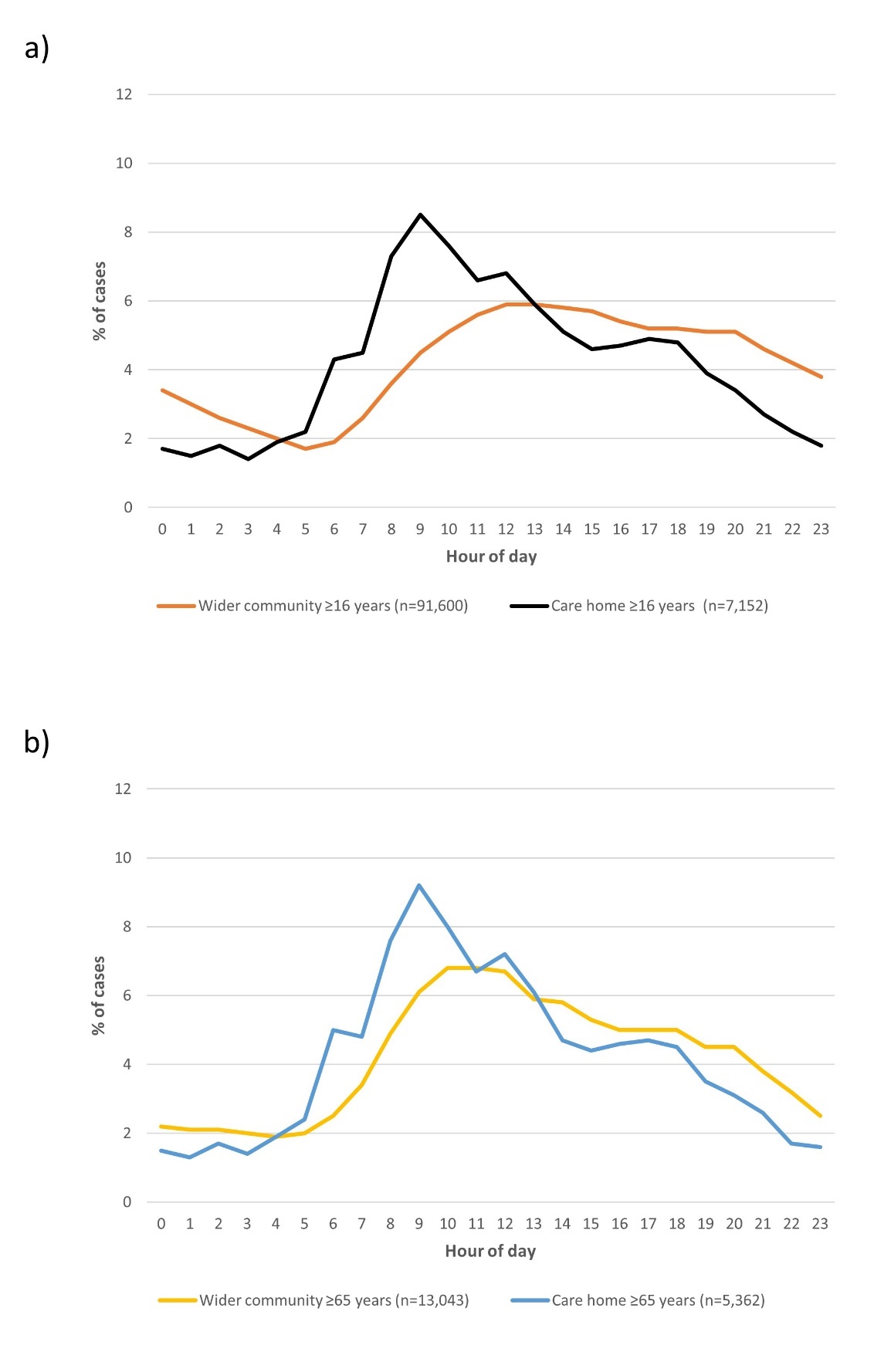


***Notes***: Hour of day reflects when call regarding the case was received. If duplicate calls were received for the same case, then earliest of the call times was selected. Exact age of persons beyond them being ≥16 years was unknown for 1,715 cases (1.7%) of cases.
