# Supplementary material for: ‘Blue-lighting’ seizure-related needs in care homes: a retrospective analysis of ambulance call-outs for seizures in North West England (2014–2021), their management and costs, with community comparisons
Source: BMJ Open. 2024 Nov 13;14(11):e089126. doi: 10.1136/bmjopen-2024-089126 (PMC11574507; doi:10.1136/bmjopen-2024-089126)
Supplement: Supplementary file 10 [file bmjopen-2024-089126.reviewer_comments.pdf]

PEER REVIEW HISTORY

BMJ Open publishes all reviews undertaken for accepted manuscripts. Reviewers are asked to complete a checklist review form and are provided with free text boxes to elaborate on their assessment. These free text comments are reproduced below.

ARTICLE DETAILS

Title (Provisional)

'Blue-lighting' seizure-related needs in care homes: A retrospective analysis of ambulance callouts for seizures in North West England (2014–2021), their management, and costs, with community comparisons

Authors

Noble, Adam J; Lane, Steven; Lloyd, Peter; Morris, Beth; Bell, Steve; Shillito, Tom; Dixon, Pete; Marson, Anthony Guy

---

VERSION 1 - REVIEW

**Reviewer**	**1**
**Name**	**Husari , Khalil S**
**Affiliation**	**Johns Hopkins University**
**Date**	**17-Jul-2024**
**COI**	**none**

The authors present retrospective data from a large regional ambulance service in the UK over 7-year period. The authors wanted to evaluate the seizure-related ambulance calls and ER transfers from care homes.

They found a higher incidence of seizure call-outs in care homes compared to general community (about 10 times). This also, increased over time from 2015 to 2022.

They also evaluate certain factors associated with ER transfers and overall related costs.

The manuscript is well written and comprehensive. Tables and figures are nicely presented. The study has significant strengths including novelty as the authors discuss and the wide geographical area/patient population (about 5.5. million).

I have the following comments:

- did the authors look into other causes of call outs? For example, stroke symptoms or AMS. If yes, were they also different between care home vs community? Do the authors thinks that the difference is related to seizures or to the practice of care homes?

- any patients adjustment between care home vs community? Patients in care homes tend to have more severe forms of epilepsy, hence this might skew the results.

- any role for acute seizure therapies (intranasal midazolam or diazepam) in decreasing the call outs?

- what is the hypothesis to explain the rising call outs in care homes?

**Reviewer**	**2**
**Name**	**Foster, Emma**
**Affiliation**	**Alfred Health, Neurology**
**Date**	**02-Oct-2024**

COI **I have no competing interests to declare in relation to this review.Please note that I/my institution has received research funding from Brain Foundation (Australia); GPCE; LivaNova (USA); Lundbeck (Australia); Monash Partners STAR Clinician Fellowship; Sylvia and Charles Viertel Charitable Foundation; The Royal Australasian College of Physicians Fellows Research Establishment Fellowship**

Thank you for writing this really interesting paper on an important research topic. It considers a) rate of ambulance call outs for seizure events, b) the proportion of these call outs that ultimately result in transfer to ED, and c) the cost of this, for care home vs adults >16 years in a region of the UK.

The research has been informed by people with lived experience in prioritising and shaping the research, further enhancing its importance and relevance for real-world settings.

The introduction is well written, and clearly lays out current knowledge gaps and the study rationale. The systematic review undertaken to inform the knowledge gaps is included at the end of the paper.

The methodology is clear, assessing practice change over large blocks of time, excluding the COVID-pandemic era.

The results are clearly laid out.

The discussion largely takes the study findings and considers important directions for future work.

I have two small points that I hope may be clarified in the manuscript (and apologies if I missed these):

1) Where did ppl get 'conveyed to', if not ED?

2) Did investigators have a feel for why patients in the Lancashire and South Cumbria regions more likely to end up in ED?

I also have two minor corrections to suggest:

1) Suggest inserting the word 'not' as needed on page 6, line 8, to be very clear, i.e., "Visiting ED after an uncomplicated seizure is not recommended, not risk-free, and not preferred [and to clarify where this preference comes from], and is costly..."

2) Suggest rephrasing sentence in 'Results / Response approach and time on scene'

"Of [delete: care home] cases involving persons aged >65..."

Thank you again for this important study.

VERSION 1 - AUTHOR RESPONSE

REVIEWER #1

COMMENT 1.1: *…The manuscript is well written and comprehensive. Tables and figures are nicely presented. The study has significant strengths including novelty as the authors discuss and the wide geographical area/patient population (about 5.5. million).*

Author response: We thank the reviewer for these positive observations.

COMMENT 1.2: *…did the authors look into other causes of call outs? For example, stroke symptoms or AMS. If yes, were they also different between care home vs community? Do the authors thinks that the difference is related to seizures or to the practice of care homes?*

Author response: The reviewer raises an interesting question – in short, why is the incidence of callouts so much higher in the care home population. We did not look at other presentations and so clues as to the answer cannot be drawn from there. Our suspicion though, is that the higher incidence is due to a combination of factors – namely, that conditions giving rise to seizures disproportionately affects older adults, that some care homes immediately call for an ambulance upon noticing a seizure (even if need might not dictate it) and because care for seizure related conditions in older adults is particularly poor which means they are more likely to experience seizures and thus have ambulance sought for them. We have introduced additional text within the manuscript accordingly. Specifically:

"The revealed incidence of seizure-related cases in care homes should be noted. The pseudo-anonymized nature of our dataset prevented us from determining how many individuals accounted for cases. However, our findings suggest a considerable number of residents necessitate specialist support. The high call-out incidence rate within the care homes population compared to the wider

population might be due to a combination of interacting factors. Namely: conditions that give rise to seizures disproportionately affect older adults; [3 4] recent qualitative work suggests a tendency in some care homes to call ambulances for suspected seizures, even when there may not be a clinical need;[40] and, finally, older adults face barriers in accessing specialist care for seizures, which may result in poorer seizure control and more frequent ambulance callouts. Further research is though, needed before definitive conclusions can be made."

COMMENT 1.3: *any patients adjustment between care home vs community? Patients in care homes tend to have more severe forms of epilepsy, hence this might skew the results.*

Author response: The nature of the dispatch data we used meant adjustments we could make were limited to age. It is possible that epilepsy might be more severe in care home residents, however, we could not make this adjustment. In view of the reviewer's helpful comment, we now make this clearer to the reader within the discussion section of our manuscript. Specifically:

"The poorer health of residents in care homes[6] might be one reason for their frequent conveyance. It is possible that paramedics encounter more individuals in care homes with abnormal observations or signs deemed worthy of ED consideration. Care home residents might even have different, more severe forms of epilepsy. However, we had limited data on the health of the individuals responsible for the call-outs and so could not adjust for health differences to test such explanations. We were able to only adjust for age. Poorer health on behalf of residents, including dementia, may also complicate paramedics ability to determine whether the person is recovering to baseline. Older persons can experience more prolonged confusion post-seizure.[17] It is noteworthy though, that paramedics did spend similar time on scene for care home and wider community cases before conveying. Paramedics indicate perceived time pressures contribute to conveyance.[23] Ambulance services might consider explicitly allowing paramedics more time for care home case assessment."

COMMENT 1.3: *- any role for acute seizure therapies (intranasal midazolam or diazepam) in decreasing the call outs??*

Author response: We agree with the reviewer that acute seizure therapies could hold potential in decreasing some need for conveyance to ED. We now discuss this in the revised manuscript. Specifically:

"Within our regression analysis, we found home type was associated with whether conveyance to ED occurred. Having accounted for reported severity, occurrence within a home specialising in learning disability (LD) reduced likelihood of ED conveyance by 35%. This might be attributable to the established association between LDs and epilepsy. Such homes might be better prepared to manage

seizures and paramedics and home staff more comfortable with individuals recovering within them. This should be explored. If the suggestion arises that staff in non-specialist homes should receive training similar to that of LD home staff, any intervention should account for potential learning decay, as ambulance cases were frequent for some homes but not for most. Forty percent in each period had at least one case.

One potentially relevant difference between care homes that do and do not specialise in LDs is the prescription of emergency rescue medication to their residents with histories of prolonged or repeated tonic-clonic seizures and the training their staff have in their use. Persons with LDs are at high risk of status epilepticus and so rescue prescriptions often considered.[48] It would be important to understand if the prescription rate for persons with similar seizures patterns in non-specialist LD homes is optimal given such treatments could permit care home staff to intervene early [49] and potentially reduce the need for conveyance to ED, even if an ambulance arrives."

COMMENT 1.4: - *what is the hypothesis to explain the rising call outs in care homes?*

Author response: This is an important question. A rise in call incidence is seen in both the care home population and in the wider community. In both populations, incidence increased by ~20% between Period I and Period IV. The reason for this is not known. During this time, the system used to code calls did not change, the epidemiology of epilepsy did not markedly change and indicators of epilepsy control (e.g., seizure freedom) within the UK population does not appear to have profoundly changed. In view of the reviewer's comments, we have revised the text to offer some potential directions for future research and wider context. Specifically:

"More detailed case data could also offer insights into the aetiology of suspected seizure cases. This might help explain the observed increase in cases which we cannot currently explain. Specifically, the incidence of suspected seizure cases inside and outside of care homes increased by around 20% between the period I and IV. This was despite NWAS' coding remaining consistent, incidence and prevalence of epilepsy not increasing [51] and seizure freedom in those diagnosed not deteriorating.[52] Moreover, the starting incidence within the region was not unusually low.[19] Previous examinations of ambulance data for other presentations in the UK and beyond have reported year-on-year increases in call numbers that cannot be satisfactorily explained by population growth.[53 54] Factors such as an increasing proportion of older adults within the population, deteriorating access to alternative services,[55] and/or a lowering of the threshold for calling an ambulances have been hypothesised as possible explanations.[56]"

REVIEWER #2

COMMENT 2.1: *Thank you for writing this really interesting paper on an important research topic…The research has been informed by people with lived experience in prioritising and shaping the research, further enhancing its importance and relevance for real-world settings…The introduction is well written, and clearly lays out current knowledge gaps and the study rationale. The systematic review undertaken to inform the knowledge gaps is included at the end of the paper…The methodology is clear, assessing practice change over large blocks of time, excluding the COVID-pandemic era….The results are clearly laid out…The discussion largely takes the study findings and considers important directions for future work.*

Author response: We thank the reviewer for these positive observations.

COMMENT 2.2: *Where did ppl get 'conveyed to', if not ED?*

Author response: In England how calls to the ambulance service are managed is typically classified according to the following categories: 'Hear & Treat', 'See & Treat', 'See & Convey to ED' and 'See & Convey Elsewhere'. The latter is a catch-all term and used to describe when an ambulance 999 call results in a face-to-face assessment of a patient by an ambulance crew at the scene and onward transport is to a health-care facility that is not an emergency department. We did not formally analyse where patients went to who received this category of care. We can though, certainly report that some patients receiving this response went to facilities suitable for low acuity cases (e.g., a GP or nurse led walk-in style urgent care centre), and some to facilities suitable for higher acuity cases (e.g., a specialist obstetrics wards, mental health units).

In view of the reviewer's comment have now more clearly noted the range of facilities where 'see & convey elsewhere' can take a patient. Specifically:

"NWAS is in many respects representative.[23] Like other English ambulance services, management options available to it are 'Hear & Treat' (telephone support only), 'See & Treat' (person is attended to by ambulance personnel but stays in place) and 'See & Convey' – be it to an emergency department (ED) ('See & Convey to ED') or an alternative healthcare facility ('See & Convey elsewhere'). The latter can include conveyance to both a low-acuity facility (e.g., general practitioner or nurse-led urgent care walk-in centres) or a potentially high-acuity facility (e.g., a specialist obstetric ward, mental health unit).[32]"

COMMENT 2.3: *Did investigators have a feel for why patients in the Lancashire and South Cumbria regions more likely to end up in ED?*

Author response: We have some suggestions that might explain the geographical variation. We have now revised the manuscript to include them as potential directions for future research. Specifically, we now state:

"Our study was unable to fully elucidate the factors influencing ED conveyance. Elsewhere [40] we report an effort to engage with care home staff for insights. Future modelling efforts may though, also consider incorporating distance between the case and ED and the extent of infrastructure in the local region that supports non-conveyance to ED (e.g., alternative destinations such as urgent care centres or access to rapid reviews by visiting healthcare professionals for residents with uncomplicated seizures). These factors may explain the geographic variations we found."

COMMENT 2.4: *Suggest inserting the word 'not' as needed on page 6, line 8, to be very clear, i.e., "Visiting ED after an uncomplicated seizure is not recommended, not risk-free, and not preferred [and to clarify where this preference comes from], and is costly...*

Author response: We have revised the sentence as per the reviewer's suggestion and included some references that support the item relating to preferences as they asked. Specifically, we now note:

"…Visiting ED after an uncomplicated seizure is not recommended,[24] not risk-free,[25 26] and is costly to the National Health Service (NHS).[19 27] Evidence from a range of sources, including discrete choice experiments, indicates such visits are also not typically preferred by with adults living with epilepsy or their significant others.[28 29]"

COMMENT 2.5: *Suggest rephrasing sentence in 'Results / Response approach and time on scene'*
"Of [delete: care home] cases involving persons aged >65..."

Author response: We have revised the sentence as per the reviewer's suggestion to:

"Of cases involving persons aged ≥65, 80.0% (CI 78.9 to 81.1) of care home cases were conveyed to ED, compared to 77.5% (CI 76.8 to 78.3) of wider community cases (Figure 1d)."

I trust that we have provided satisfactory answers to all comments by the reviewers and look forward to your response.

Yours sincerely,

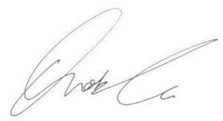

Dr. Adam Noble

University of Liverpool.
